# Identification of characteristic hub genes in the immune-active phase of chronic hepatitis B

**DOI:** 10.3389/fimmu.2026.1769749

**Published:** 2026-05-04

**Authors:** Fangfang Li, Yi Li, Yirong Du, Chao Fan, Jun Xiong, Fei Hu, Xiaoying Deng, Xiaoyan Wang, Hongyan Shi, Xiangwei Sun, Xuyang Zheng, Ying Zhang

**Affiliations:** Department of Infectious Diseases, Tangdu Hospital, The Fourth Military Medical University, Xi’an, China

**Keywords:** bioinformatics, chronic hepatitis B, immune-active phase, machine learning, solute carrier family 24 member 4

## Abstract

**Background:**

Chronic hepatitis B (CHB) caused by the hepatitis B virus (HBV) remains a major global health burden. The immune-active (IA) phase, characterized by positive Hepatitis B surface antigens (HBsAg), elevated alanine transaminase (ALT) levels, and detectable HBV DNA, is a pivotal period in the natural history of CHB. Understanding the biological process in this phase is critical for elucidating CHB pathogenesis. With the accumulation of multi-omics data, it has become feasible to identify key molecular targets regulating the host-virus interaction during the IA phase. Identifing these targets provides a critical foundation for developing novel strategies to modulate the host immune response as an important supplement to antiviral therapy.

**Methods:**

We integrated bioinformatics and machine learning approaches with clinical sample validation to identify IA-phase-specific hub genes in peripheral monocytes from CHB patients. Combining differential expression analysis and weighted gene co-expression network analysis (WGCNA), we found gene modules significantly correlated with the IA phase from the transcriptomic dataset GSE230397. IA-specific hub genes were subsequently identified using machine learning algorithms. While the potential roles of the four identified hub genes were initially explored via immune infiltration analysis, the expression of the key hub gene, solute carrier family 24 member 4 (SLC24A4), in peripheral classical monocytes from CHB patients in IA phase were specifically validated by single-cell RNA sequencing (scRNA-seq) datasets (GSE182159 and GSE283471) and confirmed by western blot analysis of clinical samples.The function of the key hub gene SLC24A4 was further explored through *in silico* knockout analysis. Structure-based virtual screening was conducted to identify potential U.S. Food and Drug Administration (FDA)-approved compounds targeting SLC24A4.

**Results:**

Compared to healthy controls (HC), 36 differentially expressed genes (DEGs) were identified as specific to the IA phase in GSE230397. WGCNA highlighted two key modules associated with IA features, from which 22 module-specific DEGs were derived. Machine learning integration pinpointed four hub genes: C-C motif chemokine receptor 5 (CCR5), interleukin 10 receptor subunit alpha (IL10RA), potassium voltage-gated channel subfamily A member 3 (KCNA3), and SLC24A4. Immune infiltration analysis of liver tissue revealed that SLC24A4 expression was positively correlated with the infiltration proportion of pro-inflammatory M1 macrophages and negatively correlated with the infiltration proportion of immunosuppressive M2 macrophages, suggesting its connection with a pro-inflammatory microenvironment. Validation in two independent scRNA-seq datasets and western blot analysis of clinical samples confirmed that SLC24A4 expression in peripheral classical monocytes was significantly higher in IA phase compared with HC. *In silico* knockout of SLC24A4 in classical monocytes using scTenifoldKnk showed the upregulation of genes involved in histocompatibility antigens class II (MHC class II)-mediated antigen presentation and the regulation of T cell activation. This suggested that SLC24A4 may act as a negative regulator of monocyte activation, and its high expression during the IA phase could represent a compensatory mechanism to restrain excessive inflammation. The possible binding of a FDA-approved compound Rucaparib to SLA24A4 was predicted by virtual screening.

**Conclusions:**

This study revealed distinct transcriptional features of the CHB IA phase and identified SLC24A4 in peripheral classical monocytes as a potential key regulator and a potential drug target for immune modulation in CHB, offering novel insights into both pathogenesis and therapeutic development.

## Introduction

Chronic hepatitis B virus (HBV) infection remains a major global health threat. It was estimated that there were 254 million prevalent hepatitis B infections and 1.1 million hepatitis B-related deaths in 2022 (1). As an important driver of liver cirrhosis and hepatocellular carcinoma, HBV profoundly impacts patient survival (2). Current treatments can effectively suppress viral replication, but achieving a sustained functional cure remains challenging largely due to an incomplete understanding of pathogenesis of chronic hepatitis B (CHB) (3, 4). The immune-active (IA) phase—characterized by detectable/elevated HBV DNA and elevated alanine transaminase (ALT)—represents a critical window of intense host-virus interaction in the natural history of CHB (5). During this phase, the immune system is activated to clear the virus but often fails, causing immunopathological damage to the liver (6). Identifying hub genes specific to the IA phase is therefore essential for elucidating its pathogenesis and exploring targets for immune modulation (7).

Advances in high-throughput sequencing have enabled integrative multi-omics analyses, providing unprecedented insights into molecular mechanisms of diseases (8). However, systematic efforts to identify and validate IA-phase-specific hub genes in CHB remain limited (9, 10). Here, we integrated bioinformatics and machine learning with clinical sample validation to address this gap. By analyzing public transcriptomic data and validating the findings in single-cell RNA sequencing (scRNA-seq) datasets as well as clinical samples, we identified solute carrier family 24 member 4 (SLC24A4) in peripheral classical monocytes as a key hub gene in patients with CHB in IA phase (in this study, peripheral classical monocytes means classical monocytes in peripheral blood). The potential immune regulatory role of SLC24A4 was further explored via *in silico* knockout and its potential as a drug target was assessed through structure-based virtual screening of U.S. Food and Drug Administration (FDA)-approved compounds. Our findings offer a novel direction for understanding immune regulation and developing therapeutic strategies for CHB.

## Materials and methods

### Retrieval of gene expression data

We searched the NCBI Gene Expression Omnibus (GEO) database using keywords such as “chronic hepatitis B” and “HBV infection”. The RNA-sequencing dataset GSE230397, which profiles liver tissue across CHB phases and healthy controls (HC), was selected for analysis (11). The inclusion and exclusion criteria for this dataset were as defined in the original publication (11). The clinical characteristics of the GSE230397 dataset can be found in the Table 1 from reference (11). Briefly, patients were stratified into the immune-tolerant (IT), immune-active (IA), inactive carrier (IC), or Hepatitis B e antigens (HBeAg)-negative hepatitis (ENEG) phases based on serum HBV DNA, ALT levels, and HBeAg status. The definition of IA phase can be found in **Supplementary Table S1** or in reference (11). All patients were antiviral treatment-naïve, had no co-existing primary liver disease, and were not co-infected with HCV, HEV, HDV, or HIV.

Additionally, the scRNA‑seq dataset GSE182159 was included for validation (12). We also conducted independent validation for SLC24A4 expression in a recently published scRNA-seq dataset GSE283471 (13), which includes paired peripheral blood mononuclear cells (PBMCs) from 3 CHB patients in IA phase along with the data of healthy controls from GSE168732. The comparison of IA-phase definitions across datasets is shown in **Supplementary Table S1** (11–13). Data were downloaded and processed using the R package GEOquery (v2.78.0) (14). Differential expression analysis was performed using the limma package (v3.66.0) (15), with results visualized via ggplot2 (v4.0.0) (16) and ComplexHeatmap (v2.26.1) (17).

### Functional enrichment analysis

Functional enrichment analyses were performed in this study, including Gene Set Enrichment Analysis (GSEA), Disease Ontology (DO), Gene Ontology (GO), and Kyoto Encyclopedia of Genes and Genomes (KEGG) enrichment analyses. GSEA was conducted using the clusterProfiler package (v4.18.1) (18) with the MSigDB gene set 'c2.cp.v7.2.symbols.gmt' (19). Significance was set at *P* < 0.10 after 10,000 permutations. GO enrichment analysis was performed on target gene sets using the clusterProfiler R package and cited the Gene Ontology Resource (20). KEGG enrichment analysis was also performed using the clusterProfiler R package and cited the KEGG database (21). Additionally, DO enrichment analysis was carried out using the DOSE R package (v4.4.0) to explore associations between hub genes and human diseases.

### Weighted gene co-expression network analysis (WGCNA)

WGCNA was applied to construct a scale-free co-expression network from the GSE230397 data (22). After sample clustering and outlier removal, a soft-thresholding power of 6 was chosen to achieve a scale-free topology. Gene modules were identified via dynamic tree cutting. Module membership (MM) and gene significance (GS) were calculated to evaluate associations between modules and clinical traits (specifically, IA phase status). Within the selected modules, genes exhibiting high module membership (MM > 0.8, indicative of high intramodular connectivity) and high gene significance (GS > 0.2, reflecting strong correlation with the IA phase) were considered as hub gene candidates.

### Identification of hub genes using machine learning

To refine IA-specific gene signatures, we employed multiple machine learning algorithms on the 22 candidate genes. The support vector machine recursive feature elimination (SVM-RFE) algorithm was used for initial feature selection based on feature importance rankings. Subsequent feature optimization was performed using least absolute shrinkage and selection operator (LASSO) regression via the glmnet package (v4.1.8) (23), applying the 1-standard error (1-SE) criterion for variable selection. A random forest (RF) algorithm was also applied. Genes were prioritized if they met algorithm-specific criteria: a top rank (SVM-RFE rank < 12), a non-zero coefficient in the LASSO model, and an importance score greater than 0.25 in the Random Forest model. The final hub genes were determined by intersecting the candidate genes identified by SVM-RFE, LASSO, and RF.

### Immune infiltration analysis and correlation analysis

To characterize the immune microenvironment during the IA phase, we selected liver tissue samples from CHB patients classified as IA within the GSE230397 datasets. Immune cell fractions were estimated from this IA group (n = 15) using CIBERSORTx (cell-type identification by estimating relative subsets of RNA transcripts, https://cibersortx.stanford.edu/) with the LM22 signature matrix (24). Spearman correlation analysis was performed between the expression of the four hub genes, including SLC24A4, C-C motif chemokine receptor 5 (CCR5), interleukin 10 receptor subunit alpha (IL10RA), potassium voltage-gated channel subfamily A member 3 (KCNA3), and immune cell infiltration proportions across these IA samples. *P*-values were adjusted for multiple testing using the Benjamini-Hochberg procedure (with FDR < 0.05 considered significant).

### Analysis of scRNA-seq data

We analyzed two independent scRNA-seq datasets—GSE182159 and GSE283471—separately. We first reanalyzed the public dataset GSE182159, which contains paired liver and peripheral blood samples from HBV-infected individuals (including 5 IA patients) and 6 HC. For this study, we focused on the peripheral blood compartment. The raw count matrix was processed using Scanpy (v1.9.6) (25) in Python (v3.10). Standard quality control filters were applied, excluding cells with fewer than 500 detected genes, more than 20,000 UMIs, or mitochondrial gene percentage exceeding 5%. After log-normalization and scaling, batch effects across multiple donors were corrected using batch balanced k nearest neighbours (BBKNN) (26). Principal component analysis was performed on highly variable genes, and cells were clustered using the Leiden algorithm (resolution = 0.6). Cell types were annotated based on canonical marker genes consistent with the original study (12), but clustering was performed independently using the Leiden algorithm. Differential expression testing between IA and HC groups within each cell type was performed using the Wilcoxon rank-sum test with Benjamini-Hochberg correction.

To independently validate the SLC24A4 expression, we analyzed a second scRNA-seq dataset (GSE283471), which comprises PBMCs from an independent dataset of CHB patients in IA phase and healthy controls (from GSE168732). No integration or joint embedding between GSE182159 and GSE283471 was performed.

### Clinical sample collection

This study was approved by the Ethics Committee of Tangdu Hospital, and written informed consent was obtained from all participants. Venous blood samples anticoagulated with EDTA were collected from CHB patients in the IA phase and from HC. IA patients were diagnosed according to the Chinese Guidelines for the Prevention and Treatment of Chronic Hepatitis B (2022 edition) (27). Inclusion criteria were Hepatitis B surface antigens (HBsAg) and HBeAg positivity, detectable HBV DNA, elevated ALT levels. The demographic and clinical characteristics of patients in the western blot validation dataset are summarized in **Supplementary Table S2**.

Although the specific ALT and HBV DNA values varied across datasets, the core definition of the IA phase remained consistent across all datasets: HBsAg positivity, elevated ALT levels, and detectable HBV DNA. Detailed IA definitions in each public dataset are provided in **Supplementary Table S1**. All included patients meet the definition of IA phase in their respective datasets.

Healthy controls for western blot validation were age-matched volunteers with no history of HBV infection or other liver diseases. Key exclusion criteria for all participants were: age <18 or >70 years; co-infection with other viruses (HCV, HEV, HDV, HIV); presence of autoimmune diseases; use of immunosuppressive or immunomodulatory medications within the past 6 months; diagnosis of malignancies; presence of severe or uncontrolled concurrent medical conditions, including but not limited to cardiovascular diseases, diabetes mellitus, chronic kidney disease, and significant hematological disorders; pregnancy or lactation; and unwillingness to participate. PBMCs were isolated via Ficoll density gradient centrifugation.

### Monocyte isolation and western blot validation

Classical monocytes were isolated from PBMCs by immunomagnetic selection using the EasySep™ Human Monocyte Isolation Kit (CD14^+^CD16^-^, STEMCELL Technologies, Cat# 19359) according to the manufacturer’s instructions.

The purity of the isolated CD14^+^CD16^-^ monocytes was assessed by flow cytometry. Cells were stained with fluorochrome-conjugated antibodies against CD14 (PerCP/Cyanine5.5 anti-human CD14, BioLegend, Cat# 325622) and CD16 (APC/Cyanine7 anti-human CD16, BioLegend, Cat# 302018). Corresponding isotype controls (PerCP/Cyanine5.5 Mouse IgG1, κ Isotype Ctrl, BioLegend, Cat# 400150; and APC/Cyanine7 Mouse IgG1, κ Isotype Ctrl, BioLegend, Cat# 400128) were used. Stained cells were analyzed on a Beckman Coulter CytoFLEX flow cytometer (Beckman Coulter, Inc., Brea, CA, USA), and data were analyzed using FlowJo software (TreeStar, Ashland, OR, USA). We gated monocytes based on FSC/SSC characteristics. We also conducted doublet exclusion and live cell gating (using Zombie Aqua™ Fixable Viability Dye, BioLegend, Cat# 423102). CD14/CD16 gating was conducted. CD45^+^ cells were gated in advance using CD45 antibody (CD45 monoclonal antibody, FITC, eBioscience, Cat# 11-0459-42) and corresponding isotype control (Mouse IgG1 kappa Isotype Control, FITC, eBioscience, Cat# 11-4714-82).

Purified classical monocytes were lysed in RIPA buffer containing protease inhibitors. Protein samples (20 µg per lane) were separated by SDS-PAGE, transferred to PVDF membranes, and blocked with 5% non-fat milk. For SLC24A4 detection, membranes were incubated overnight at 4 °C with primary antibody against SLC24A4 (1:1000, Proteintech Cat# 18992-1-AP), followed by HRP-conjugated Goat Anti-Rabbit IgG (H+L) secondary antibody (1:5000, Proteintech Cat# SA00001-2) for 1 h at room temperature. Protein bands were visualized using an enhanced chemiluminescence (ECL) reagent (Bio-Rad, Cat# 1705060). After detection, the membrane was incubated in western blot stripping Buffer (Thermo Fisher Scientific, Cat# 46430) for 15 min at room temperature to remove the bound antibodies. Following extensive washing and re-blocking, the membrane was reprobed with an HRP-conjugated β-actin antibody (1:5000, Proteintech Cat# HRP-66009) as a loading control. Protein bands were quantified using ImageJ software (v1.54) (28). SLC24A4 expression levels were normalized to β-actin for each sample.

### *In silico* knockout analysis using ScTenifoldKnk

To infer the functional impact of SLC24A4 on the immune response to HBV, we performed *in silico* gene knockout using scTenifoldKnk (v1.0.3) (29). Single-cell count matrices were extracted from classical monocytes isolated from the Blood_IA subset of GSE182159, as annotated by canonical surface markers (e.g., high CD14 and low/negative FCGR3A/CD16 expression). Lymphocytes were explicitly excluded from the analysis to ensure cell-type-specific inference. The top 1,000 highly variable genes were selected within the classical monocyte compartment using the variance stabilizing transformation (vst) method, and SLC24A4 was forcibly included in the gene set regardless of its variability. To balance computational efficiency and biological representation, we subsampled the 1,000 cells with the highest total UMI counts. Differential gene regulation between the wild-type and virtual-knockout manifolds was assessed using the built-in statistical framework of scTenifoldKnk, and genes with an adjusted *P*-value < 0.05 were considered significantly dysregulated. Downstream pathway enrichment analysis was performed using clusterProfiler (v4.18.1) against the GO and KEGG databases.

### Virtual screening for potential drug candidates

To identify FDA-approved compound possibly modulating SLC24A4 activity, we performed structure-based virtual screening. The three-dimensional structure of SLC24A4 (UniProt ID: Q8NFF2) (30) was predicted using AlphaFold2 (31) via the ColabFold pipeline (32). Model quality was assessed using ProSA-web (33), which calculates a Z-score reflecting the overall structural reliability by comparing the model with experimentally determined structures deposited in the Protein Data Bank (PDB). A library of 1,615 approved compounds was retrieved from the ZINC20 database (34). Their 3D conformations were generated and energy-minimized using Open Babel (35). Molecular docking was performed using AutoDock Vina (36), with the single AlphaFold-predicted structure of SLC24A4 serving as the receptor. The binding site was defined based on the transmembrane cavity identified by structural analysis (P_0; drug score 0.81, volume 2578.26 Å³; see **Supplementary Table S3**). From the initial library, the top 10 compounds with the highest binding affinities (lowest kcal/mol) were selected as potential hits. The protein-ligand interaction modes of these top 10 candidates were visualized using PyMOL (Schrödinger, LLC, New York, NY). A *P*-value (or adjust *P*-value) < 0.05 was considered statistically significant unless otherwise specified in this article. R (version 4.5.1, R Foundation for Statistical Computing) was used for statistical analysis if not stated otherwise in this article.

## Results

### IA phase exhibited a unique set of differentially expressed genes (DEGs)

**Figure 1:**
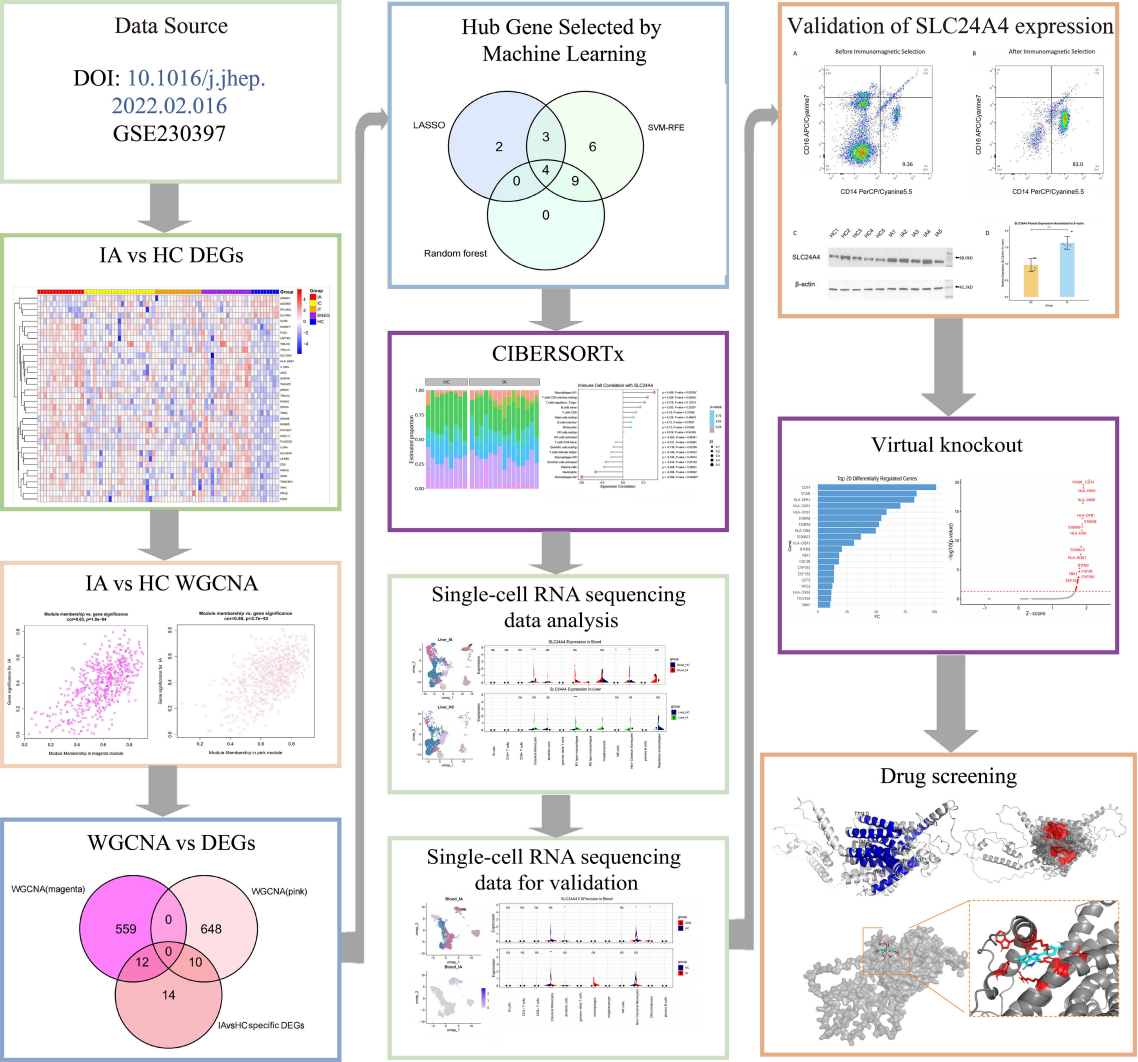
Flowchart of the analysis and validation strategy. IA, immune-active; HC, healthy controls; DEGs, differentially expressed genes; WGCNA, weighted gene co-expression network analysis; FDA, U.S. Food and Drug Administration; CIBERSORTx, cell-type identification by estimating relative subsets of RNA transcripts; M1, classically activated macrophages; M2, alternatively activated macrophages.

**Figure 2:**
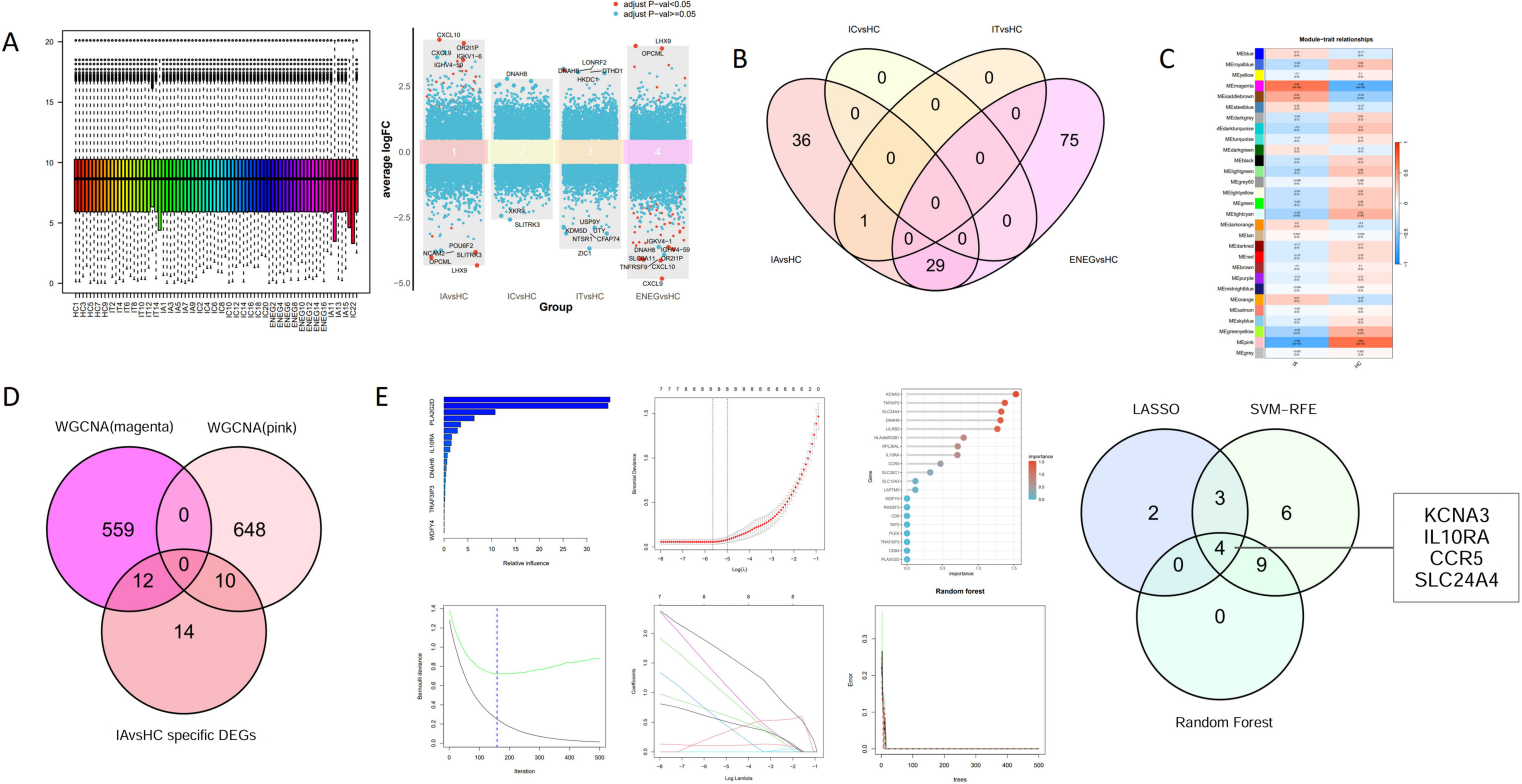
Identification of differentially expressed genes (DEGs) and candidate hub genes. **(A)** Box plots of normalized raw data across samples and multigroup volcano plots of DEG expression from four comparison groups (IA vs HC, IC vs HC, IT vs HC, and ENEG vs HC). **(B)** Venn diagram of different comparisons between groups in GSE230397. There were 36 specific DEGs in IA vs HC, which were unchanged in other comparisons. **(C)** Module-trait heatmap showing correlation between gene modules and IA phase. Color represents correlation coefficient, the number in each grid indicates the correlation coefficient and *P*-value (*P*-value is in parentheses). The magenta module (positively correlated) and pink module (negatively correlated) were selected for further analysis. **(D)** Venn diagram showing the overlap between IA-specific DEGs (n = 36) and genes from the magenta and pink WGCNA modules, yielding 22 candidate genes for machine learning prioritization. **(E)** Feature selection using three algorithms: support vector machine recursive feature elimination (SVM-RFE), least absolute shrinkage and selection operator (LASSO), and Random Forest (RF). Venn diagram showing the four hub genes obtained from the intersection of the results from the SVM-RFE, RF, and LASSO algorithms. IT, immune-tolerant; IA, immune-active; IC, inactive carrier; ENEG, HBeAg-negative hepatitis; HC, healthy controls; WGCNA, weighted gene co-expression network analysis; SVM-RFE, support vector machine recursive feature elimination; LASSO, least absolute shrinkage and selection operator.

The research workflow is illustrated in **Figure 1**, it commenced with data preprocessing and normalization of the GSE230397 dataset. Quality control was rigorously performed by comparing expression distributions before and after normalization, as shown in **Supplementary Figure S1A** (raw data) and **Supplementary Figure S1B** (normalized data). Sample clustering and principal component analysis (PCA) are in **Supplementary Figures S1C, D**. Subsequently, differential expression analysis was conducted across four comparison groups. **Figure 2A** displays the box plots of the final normalized data alongside multigroup volcano plots for the comparisons: IA vs. HC, IC vs. HC, IT vs. HC, and ENEG vs. HC. The complete list of 36 IA-specific DEGs is provided in **Supplementary Table S4**. Among these 36 DEGs, 22 were upregulated and 14 were downregulated. A Venn diagram illustrating the uniqueness of these 36 IA-specific DEGs across the four comparison groups is shown in **Figure 2B**. GSEA performed on all significantly upregulated genes in the IA group revealed significant enrichment in some immune-related pathways (e.g., allograft rejection, graft-versus-host disease), while downregulated genes were enriched in some metabolism-related pathways (e.g., drug metabolism-cytochrome P450, primary bile acid biosynthesis) (**Supplementary Figure S2)**.

### WGCNA revealed two gene modules strongly correlated with the IA phase

After quality control (**Supplementary Figures S1C, D**), 9 HC and 15 IA samples from GSE230397 were used for WGCNA. Sample clustering, soft threshold details, and the module dendrogram are shown in **Supplementary Figure S3**. Correlation analysis between module eigengenes and clinical traits identified the magenta module as the most positively correlated with the IA phase and the pink module as the most negatively correlated (**Figure 2C**). Further characterization of the identified modules, including the enlarged module-trait correlation heatmap, clustering dendrogram of module eigengenes, and network heatmap of selected modules, is provided in **Supplementary Figure S4**. A Venn diagram showing the overlap between IA-specific DEGs and genes from the magenta and pink WGCNA modules, which yielded 22 hub gene candidates as presented in **Figure 2D**. DO analysis linked these WGCNA-filtered IA-specific hub gene candidates to specific autoimmune and infectious conditions. As shown in **Supplementary Figures S5A, B**, the enriched diseases included multiple sclerosis, psoriasis, systemic juvenile rheumatoid arthritis, anterior uveitis, and panuveitis, as well as infectious diseases such as pulmonary tuberculosis and primary bacterial infectious disease. GO enrichment analysis further elucidated their functional roles. In terms of Biological Processes (BP), these genes were significantly involved in the regulation of leukocyte cell-cell adhesion and the regulation of T cell activation (**Supplementary Figure S5C**). Molecular Function (MF) analysis highlighted their capacity for immune receptor activity, histocompatibility antigens (MHC) protein complex binding and peptide antigen binding (**Supplementary Figure S5D**). Consistent with these findings, KEGG pathway analysis revealed significant associations with pathways including Epstein-Barr virus infections, Human cytomegalovirus infections, Virion - Human immunodeficiency virus, antigen processing and presentation, and T cell (Th1, Th2, and Th17) differentiation (**Supplementary Figures S6A, B**).

### Integration of machine learning pinpointed four IA-specific hub genes

Using the 22 WGCNA-filtered IA-specific hub gene candidates, we applied three machine learning-based feature selection algorithms with the criteria described in Methods. The intersection of genes meeting all three criteria yielded four robust hub genes: CCR5, KCNA3, SLC24A4, and IL10RA (**Figure 2E**). The Module Membership (MM) and Gene Significance (GS) values for the 22 candidate genes identified in the magenta and pink modules are provided in **Supplementary Table S5**. The *P*-value for GS indicates the significance of the correlation between gene expression and the IA trait. The full list of 22 candidate genes and their machine learning importance metrics are provided in **Supplementary Tables S5**, **6**, respectively. All four hub genes exhibited significantly higher expression in the IA group compared to healthy controls (**Supplementary Figures S7A–D**). GSEA revealed that high expression of these hub genes was associated with enrichment of some immune-related pathways, while low expression correlated with some metabolic pathways (**Supplementary Figures S7E-H** and **S8A-D**).

### Hub genes expression correlated with macrophage polarization in the liver

**Figure 3:**
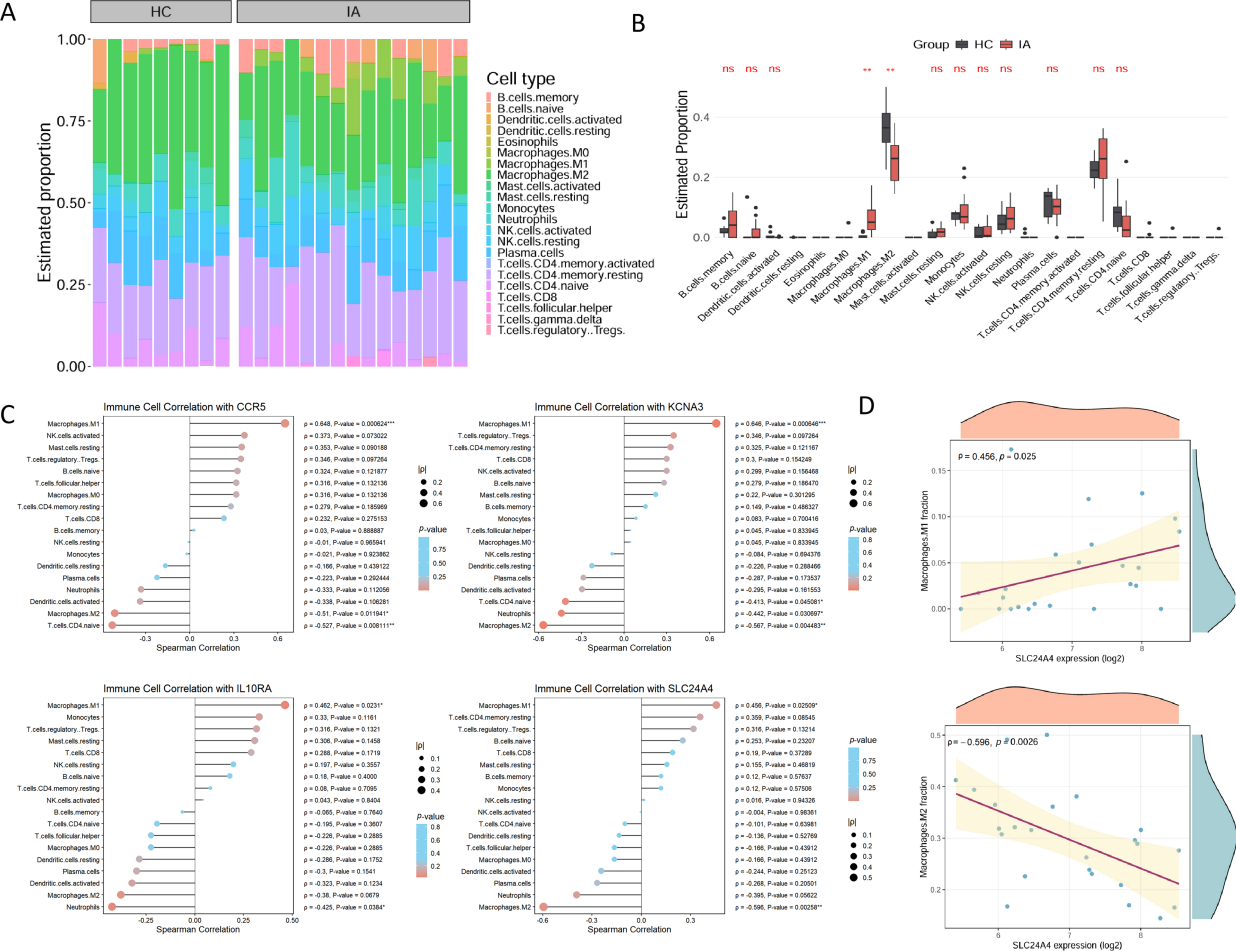
CIBERSORTx deconvolution links hub gene expression to intrahepatic immune landscape in IA-CHB. **(A)** Immune cell composition in liver tissue of IA patients (n = 15) and healthy controls (HC, n = 9). Stacked bar plot shows the estimated proportions of 22 immune cell types inferred by CIBERSORTx. **(B)** Box plots comparing the estimated proportions of all 22 immune cell types in liver tissue between IA (n = 15) and HC (n = 9) groups. Cell types with statistically significant differences (Wilcoxon rank-sum test) are indicated with asterisks: **P<0.01; ns: not significant (*P*≥0.05); no marker indicates that the Wilcoxon rank-sum test can not be performed (e.g., due to zero proportion across all samples or insufficient sample size for that cell type). **(C)** Spearman correlation heatmap between the expression of four hub genes (SLC24A4, CCR5, IL10RA, KCNA3) and the infiltration proportions of immune cell types. For clarity, only cell types with detectable abundance and computable correlation coefficients are displayed; cell types with negligible abundance are excluded from the heatmap. Color intensity represents the correlation coefficient (ρ). Asterisks indicate statistically significant correlations (*P<0.05, **P<0.01, ***P<0.01). A complete list of correlation results for all 22 immune cell types is provided in **Supplementary Table S7**. **(D)** Scatter plots depicting the correlation between SLC24A4 expression and the infiltration proportion of M1 macrophages (up) and M2 macrophages (down). Spearman’s ρ and *P*-values are shown. SLC24A4 expression was positively correlated with M1 macrophages (ρ = 0.456, *P*-value = 0.025) and negatively correlated with M2 macrophages (ρ = -0.596, *P*-value = 0.0026). CIBERSORTx, cell-type identification by estimating relative subsets of RNA transcripts; IA, immune-active; HC, healthy controls; M1, classically activated macrophages; M2, alternatively activated macrophages.

To investigate the immune implications of the four hub genes, we performed CIBERSORTx deconvolution on the bulk liver transcriptomic dataset GSE230397. The inferred immune cell fractions revealed a significant increase in M1 macrophages in IA patients compared to healthy controls (**Figures 3A, B**). The overall composition of 22 immune cell subtypes is shown in **Supplementary Figure S9A**, while significant differences between IA and HC groups are detailed in **Supplementary Figure S9B**. Spearman correlation analysis between hub gene expression and immune cell infiltration proportions showed that SLC24A4 expression was significantly associated with macrophage polarization; specifically, it was positively correlated with the infiltration proportion of pro-inflammatory M1 macrophages (ρ = 0.456, *P*-value = 0.02509) and negatively correlated with the infiltration proportion of immunosuppressive M2 macrophages (ρ= -0.596, *P*-value = 0.00258) (**Figures 3C, D**; detailed statistics in **Supplementary Table S7**). Detailed statistics for the four hub genes are in **Supplementary Table S7**, different ρ and *P*-values in different graphs for the same gene are due to different decimal places retained). CCR5 and KCNA3 exhibited similar dual correlations, showing significant positive associations with M1 and negative associations with M2 macrophages. In contrast, IL10RA was significantly correlated only with M1 macrophages (*P*< 0.05), with no statistically significant association observed for M2 macrophages (*P* > 0.05) (**Figure 3C**, **Supplementary Table S7**). Notably, correlation coefficients for certain cell types could not be reliably estimated due to their very low or undetectable abundance in the liver tissue samples and are therefore not displayed in **Figure 3C**. The complete correlation results for all 22 immune cell types are available in **Supplementary Table S7**. Additional details and scatter plots illustrating the correlations of CCR5, KCNA3, and IL10RA with M1/M2 macrophages are provided in **Supplementary Figures S9C, D**).

Given that hepatic macrophages are predominantly derived from circulating monocytes (37), and scRNA-seq analysis confirmed that SLC24A4 was specifically upregulated in classical monocytes during the IA phase (see below), these findings suggest that SLC24A4-high monocytes may be predisposed to differentiate into M1-like pro-inflammatory macrophages upon liver infiltration, thereby shaping a permissive microenvironment for T cell activation and contributing to the immune pathogenesis of the IA phase.

### scRNA-seq validation confirmed the high expression of SLC24A4 in peripheral classical monocytes during the IA phase

Although bulk liver tissue analysis provided critical insights into the hepatic immune microenvironment, the invasive nature of liver biopsies limits their utility for routine monitoring. We therefore investigated whether the identified hub gene SLC24A4 exhibited consistent expression patterns in peripheral blood immune cells. To this end, we analyzed two independent scRNA-seq datasets (GSE182159 and GSE283471). Immune cell subpopulations, particularly classical monocytes, were identified based on standard marker genes (**Supplementary Table S8**).

**Figure 4:**
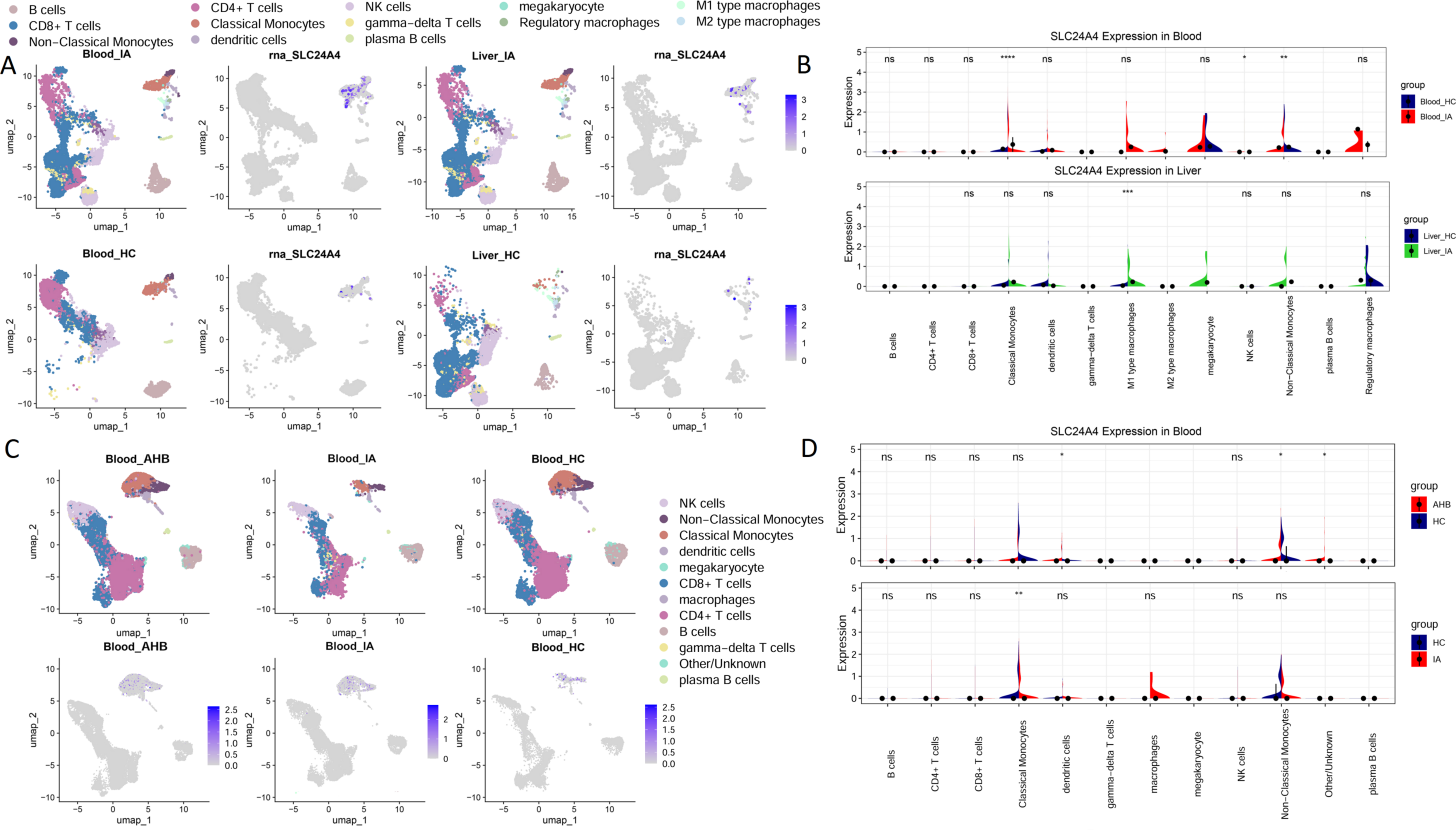
SLC24A4 expression was significantly higher in classical monocytes from IA patients compared to HC. **(A)** UMAP visualization of GSE182159 PBMCs, with classical monocytes annotated. **(B)** Violin plot of SLC24A4 expression in classical monocytes across datasets in GSE182159, comparing IA vs HC. **(C)** UMAP replication in GSE283471 blood dataset, with monocytes annotated across IA vs HC and AHB vs HC groups. **(D)** Violin plot of SLC24A4 expression in classical monocytes across datasets in GSE283471, comparing IA vs HC and AHB vs HC. UMAP, uniform manifold approximation and projection; PBMCs, peripheral blood mononuclear cells; IA, immune-active; HC: healthy control; AHB, acute hepatitis B. Statistical significance was determined using the Wilcoxon rank-sum test. *P<0.05, **P<0.01, ***P<0.001, ****P<0.0001; ns, not significant (*P*≥0.05); no marker indicates that the gene’s expression level is constantly 0 in this cell type, making it impossible to perform the Wilcoxon rank-sum test.

In the GSE182159 dataset, SLC24A4 expression was significantly higher in classical monocytes from the IA phase compared with HC (**Figures 4A, B**). This finding was replicated in the GSE283471 dataset (**Figure 4C, D**). Notably, GSE283471 dataset included an acute hepatitis B (AHB) group, in which SLC24A4 expression in classical monocytes showed no significant difference compared to HC (**Figures 4C, D**). Details about AHB group can be seen in reference (13).

### Significantly higher expression of SLC24A4 protein in IA patients compared to HC

**Figure 5:**
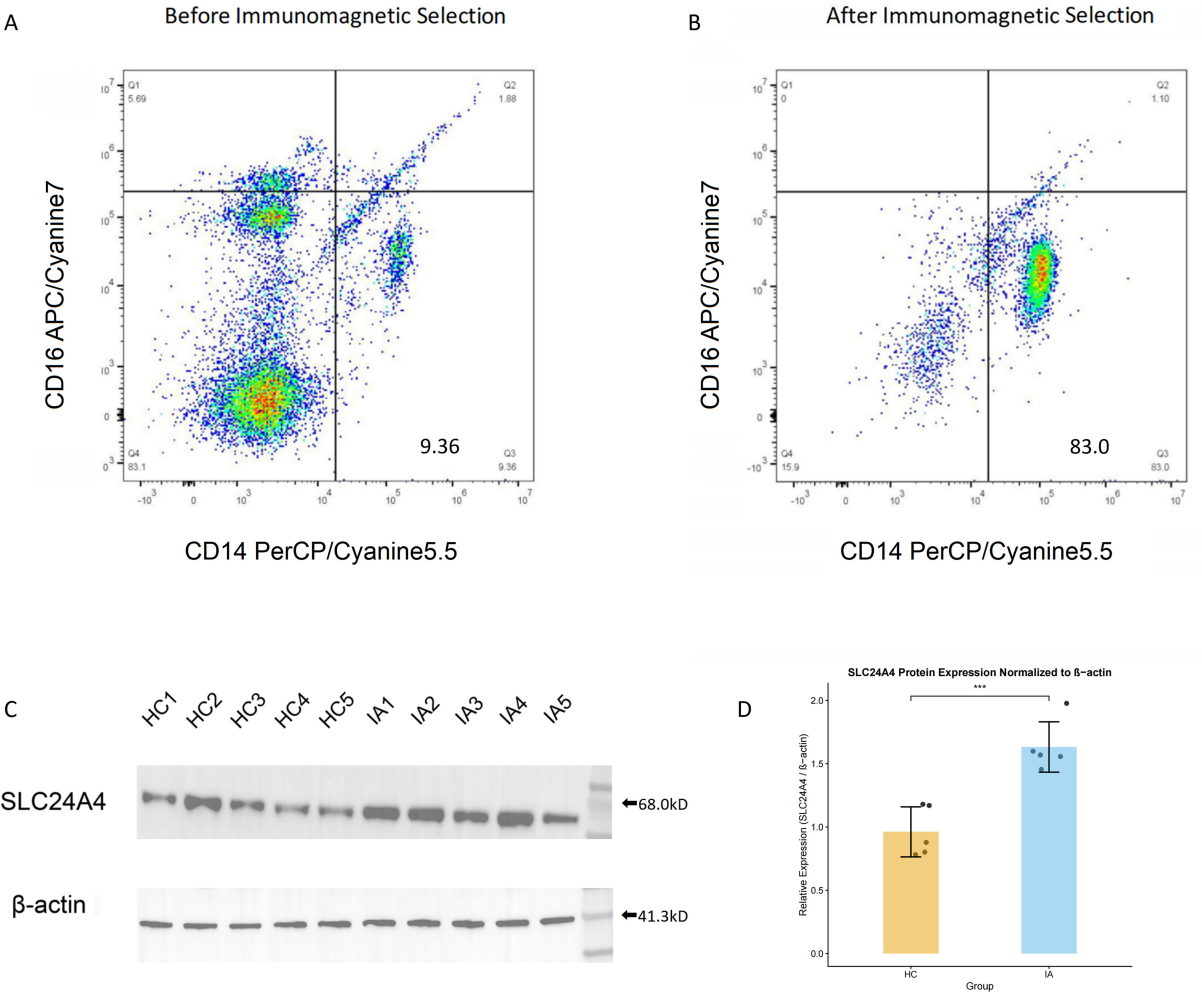
Western blot analysis of SLC24A4 protein expression in CD14^+^CD16^-^ monocytes. Flow cytometric assessment of classical monocyte (CD14^+^CD16^−^) purity before **(A)** and after **(B)** immunomagnetic selection. **(C, D)** Western blot (WB) analysis of SLC24A4 in sorted monocytes from 5 IA patients and 5 HC donors. C: Blots for SLC24A4 and β-actin (loading control). D: Quantification of SLC24A4 normalized to β-actin (n = 5 biological replicates per group). Data are shown as individual values with median and interquartile range (IQR); ***P < 0.001 by Mann-Whitney U-test. Uncropped blots are available in **Supplementary Figure S10**. IA, immune-active; HC, healthy controls; WB, Western blot.

To confirm our bioinformatic findings, we isolated classical monocytes from PBMCs of 5 IA patients and 5 HC (**Figures 5A, B**). Western blot analysis demonstrated that SLC24A4 protein expression normalized by β-actin was significantly higher in classical monocytes from IA patients compared to HC (**Figures 5C, D**). Uncropped western blot images are available in **Supplementary Figure S10A** (SLC24A4) and S10B (β-actin).

### *In silico* knockout of SLC24A4 enhanced pro-inflammatory and antigen-presenting programs in monocytes

**Figure 6:**
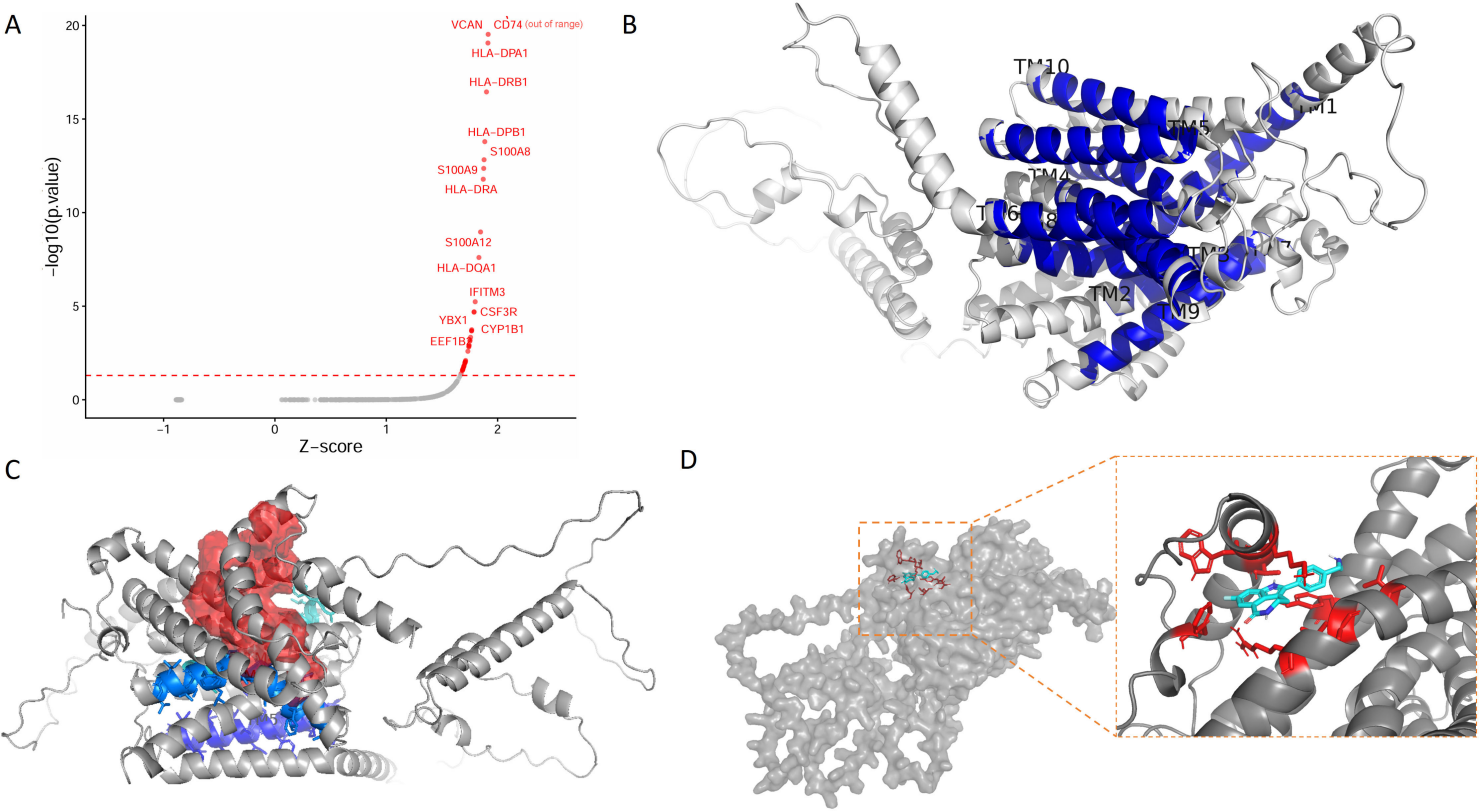
SLC24A4 is a structurally druggable target whose inhibition predicted anti-inflammatory effects. **(A)** Volcano plot showing significantly upregulated genes upon SLC24A4 knockout in classical monocytes. **(B)** AlphaFold2-predicted structure of SLC24A4 with 11 transmembrane helices (TM1–TM11) annotated based on TMHMM. **(C)** Druggable pocket predicted by DoGSiteScorer (drug score = 0.81, volume = 2578.26 Å³), located in the central cavity of the transmembrane domain, representing the predicted ion translocation pathway of this K^+^-dependent Na^+^/Ca2^+^ exchanger. The pocket is shown as a red transparent isosurface (contour level = 1.0); transmembrane helices are shown as ribbons with TM3 (cyan), TM4 (blue), and TM5 (purple) highlighted as key contributors to the pocket lining. **(D)** Predicted binding pose of Rucaparib within the druggable pocket. log2FC, log2 fold change; TM, transmembrane; TMHMM, transmembrane hidden Markov model; DoGSiteScorer, a tool for pocket detection and druggability assessment.

To assess the functional role of SLC24A4 in HBV-induced immune response, we performed *in silico* knockout using scTenifoldKnk exclusively in classical monocytes from the Blood_IA dataset (GSE182159). Virtual ablation of SLC24A4 significantly upregulated genes encoding histocompatibility antigens class II (MHC class II) components (CD74, FC = 101.9; major histocompatibility complex, class II, DR alpha, HLA-DRA, FC = 49.9; major histocompatibility complex, class II, DR beta 1, HLA-DRB1, FC = 71.0), inflammatory alarmins (S100 calcium binding protein A8, S100A8, FC = 54.6; S100 calcium binding protein A9, S100A9, FC = 52.5), and the extracellular matrix modulator versican (VCAN, FC = 85.0) (**Figure 6A**; **Supplementary Figure S11A**; all adjusted *P* < 0.05).

Pathway enrichment analysis revealed that these upregulated genes were most significantly enriched in the Gene Ontology term “peptide antigen assembly with MHC class II protein complex” (GO:0002399, adjusted *P* = 2.94 × 10^-13^). Concordantly, KEGG analysis highlighted significant enrichment in the “Antigen processing and presentation” pathway (hsa04612, adjusted *P* = 2.28 × 10^-11^), which encompasses the core MHC class II machinery. Additional pathways, including “Phagosome” (hsa04145) and “Hematopoietic cell lineage” (hsa04640), further supported a shift toward an activated, immunostimulatory monocyte state (**Supplementary Figures S11B, C**). Collectively, these results indicate that SLC24A4 functions as a transcriptional brake on monocyte activation; its loss unleashes a robust antigen-presenting and pro-inflammatory program.

### Virtual screening identified the possible binding of rucaparib to SLC24A4

To explore the potential as a drug target of SLC24A4, we conducted structure-based virtual screening against a curated library of FDA-approved drugs. The AlphaFold2-predicted structure of SLC24A4, with its 11 transmembrane helices annotated, was shown (**Figure 6B**). The overall quality of the predicted structure was assessed using ProSA-web, with the Z-score falling within the range characteristic of native protein structures of similar size (**Supplementary Figure S12A**). DoGSiteScorer analysis identified multiple potential binding pockets in the SLC24A4 structure (**Supplementary Table S3**). The largest and most prominent pocket, P_0, occupies the central cavity of the transmembrane domain and likely corresponds to the ion translocation pathway of this K^+^-dependent Na^+^/Ca^2+^ exchanger (**Figure 6C**). With a drug score of 0.81 and a volume of 2578.26 Å³, this site was selected for virtual screening of FDA-approved compounds. Using AutoDock Vina, we found Rucaparib, a clinically approved poly(ADP-ribose) polymerase (PARP) inhibitor, emerged as the top hit with strong predicted binding affinity (−8.78 kcal/mol). The predicted binding pose of the top hit (Rucaparib) was shown (**Figure 6D**). The details for the top 10 hits are listed in **Supplementary Table S9**. The docking poses for the top 10 candidates are provided in **Supplementary Figures S12B–K**. These findings positioned Rucaparib as a promising repurposing candidate for targeting SLC24A4.

## Discussion

SLC24A4, also known as NCKX4, is a K^+^-dependent Na^+^/Ca^2+^ exchanger that facilitates Ca^2+^ extrusion, thereby maintaining intracellular calcium homeostasis (38).While its most well-characterized roles are in sensory systems (39, 40) and particularly in dental enamel formation—where it localizes to the apical membrane of maturation-stage ameloblasts and is essential for proper mineralization (41–43). Recent database analyses indicate that SLC24A4 may have previously unrecognized roles in various disease contexts, including cancer and viral infection. In hepatocellular carcinoma (HCC), The Cancer Genome Atlas (TCGA) Program data revealed sex-dimorphic epigenetic modifications in SLC24A4, such as promoter hypomethylation in males and gene-body hypermethylation in females, suggesting potential context-dependent regulation (44). Furthermore, the Virus Mutations, Integration sites and cis-effects (ViMIC) database identified SLC24A4 as a target gene of human T-lymphotropic virus 1 (HTLV-1), with its expression correlating with immune infiltration in HTLV-1-associated diseases (45). These findings imply a broader role of SLC24A4 in virus-host immune interactions. However, the function of SLC24A4 in hepatocarcinogenesis, viral hepatitis, and immune-mediated liver injury remain largely unexplored.

CHB progresses through distinct immunological phases, with the IA phase representing a critical window of intense host–virus interaction, marked by elevated ALT, hepatic inflammation, and increased risk of cirrhosis and HCC. While adaptive immune dysfunction—particularly T-cell exhaustion—has been well documented, the molecular drivers of innate immune activation during this phase remain to be defined. Leveraging an integrative bioinformatics pipeline combining differential expression analysis, WGCNA, and machine learning on liver transcriptomes (GSE230397), we identified four hub genes consistently upregulated in the IA phase: CCR5, IL10RA, KCNA3, and SLC24A4. Among these, SLC24A4 showed reproducible elevation in peripheral classical monocytes across two independent scRNA-seq datasets (GSE182159 and GSE283471), further confirmed by western blot analysis in sorted classical monocytes from IA patients. This positions SLC24A4 as a uniquely accessible immune-associated marker.

Deconvolution of bulk liver data revealed that SLC24A4 expression was positively correlated with the infiltration proportion of M1 macrophages and negatively correlated with the infiltration proportion of M2 macrophages suggesting a link to pro-inflammatory polarization. Paradoxically, *in silico* knockout analysis indicated that SLC24A4 may act as a transcriptional repressor of antigen presentation, as its virtual ablation led to the upregulation of MHC class II genes. We reconcile these observations by proposing that high expression of SLC24A4 in classical monocytes in IA-phase represents a compensatory negative feedback mechanism—an attempt to restrain excessive activation and limit immunopathology in the face of sustained inflammatory stimuli. However, the inflammatory response still overwhelms the regulatory effort of SLC24A4. This hypothesis is consistent with the established role of SLC24A4 in Ca^2+^ homeostasis. Dysregulated Ca^2+^ signaling can activate the NLRP3 inflammasome and lead to cytokine release (46, 47). Thus, SLC24A4 may function as a critical buffer against Ca^2+^ overload during immune activation. Further investigation is needed to determine whether it operates exclusively in the forward mode (Ca^2+^ extrusion) or can also operate reversibly under specific electrochemical conditions (48, 49).

As a membrane ion transporter, SLC24A4 is potentially druggable (50). Using the AlphaFold2-predicted structure of SLC24A4, we performed virtual screening of FDA-approved compounds against its central cavity and identified Rucaparib—a clinically approved PARP inhibitor—as a top candidate with strong predicted binding affinity (-8.78 kcal/mol) to this site (51). Interestingly, PARP inhibitors possess intrinsic immunomodulatory properties, primarily through activation of the cGAS-STING pathway (52, 53). This raises the intriguing possibility that if Rucaparib indeed binds to SLC24A4. We speculate that the effect of Rucaparib in CHB could involve a dual mechanism: direct modulation of the proposed SLC24A4-mediated feedback brake in monocytes, and indirect immunomodulation via PARP inhibition. However, these findings are purely computational and require experimental validation. Future studies should employ CRISPRi knockdown, live-cell Ca^2+^ imaging, and functional assays in primary human monocytes to define the function of SLC24A4 in the immunopathogenesis of CHB. Additionally, electrophysiological approaches and ion imaging may help clarify the Ca^2+^-handling mechanisms of SLC24A4 in the context of viral infection (54, 55).

In conclusion, our multi-omics approach identifies SLC24A4 as a novel regulator of monocyte function during the IA phase of CHB. Its consistent upregulation in both hepatic and peripheral compartments—and its potential role in balancing inflammation and immune restraint—highlights SLC24A4 as a promising biomarker and a potentially druggable therapeutic target.

## Data Availability

The datasets presented in this study can be found in online repositories. The names of the repository/repositories and accession number(s) can be found in the article/**Supplementary Material**. Publicly available data used in this study can be found in their respective data sources. Further information for this study are available from the corresponding authors on request.
