# Supplementary material for: Identification of characteristic hub genes in the immune-active phase of chronic hepatitis B
Source: Front Immunol. 2026 May 4;17:1769749. doi: 10.3389/fimmu.2026.1769749 (PMC13180856; doi:10.3389/fimmu.2026.1769749)
Supplement: Supplementary file 1 [file Supplementaryfile1.docx]

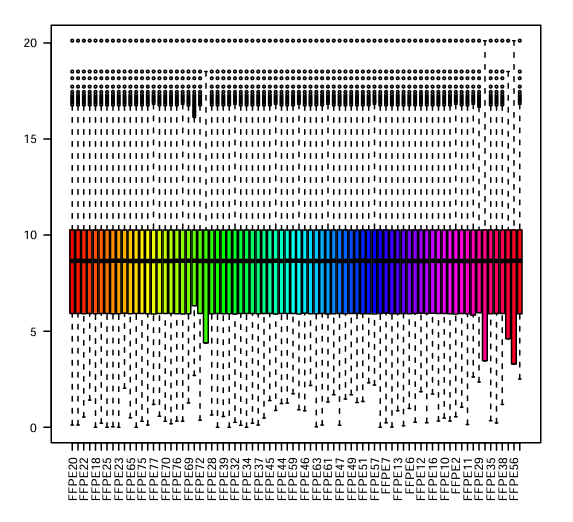

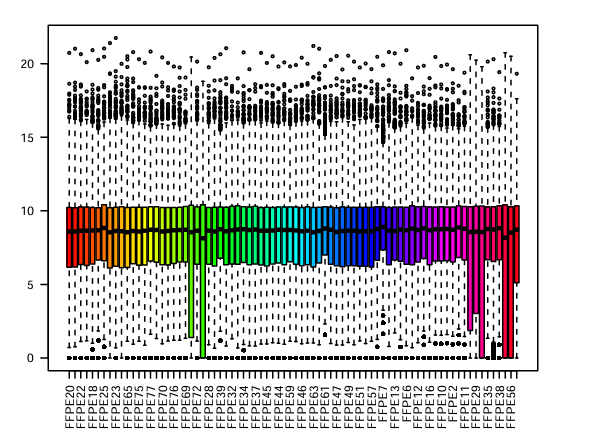


A

B


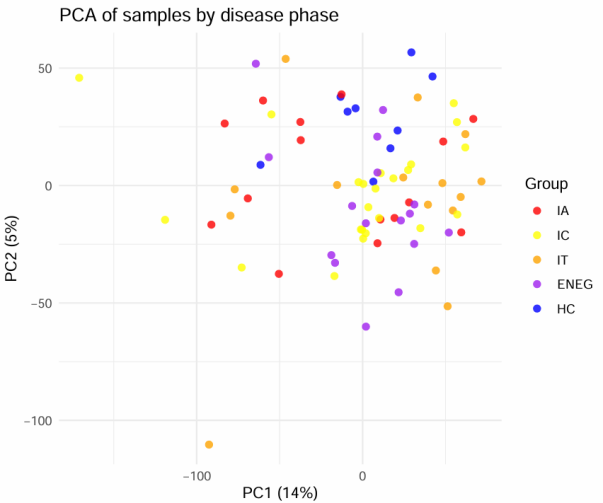

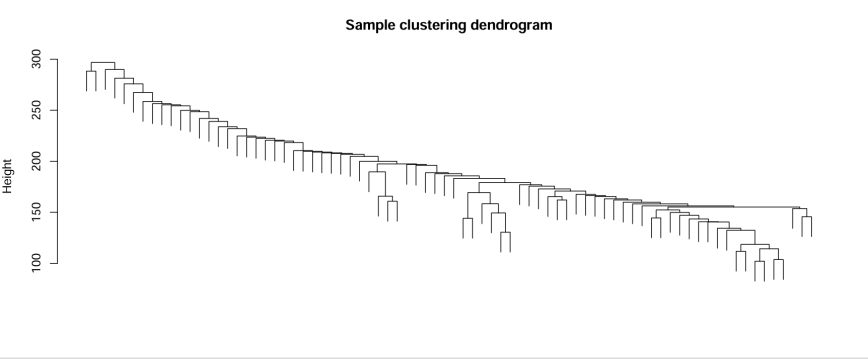


D

C

**Figure S1** | Quality control and preprocessing of GSE230397 dataset

A: Boxplot of raw expression values across samples (FFPE, Formalin fixed paraffin embedded, the relationship between FFPE number and the samples can be seen in reference 11,)

B: Boxplot of normalized expression values across samples.

C: Sample clustering dendrogram for outlier detection.

D: Principal component analysis (PCA) showing sample distribution by disease phase.


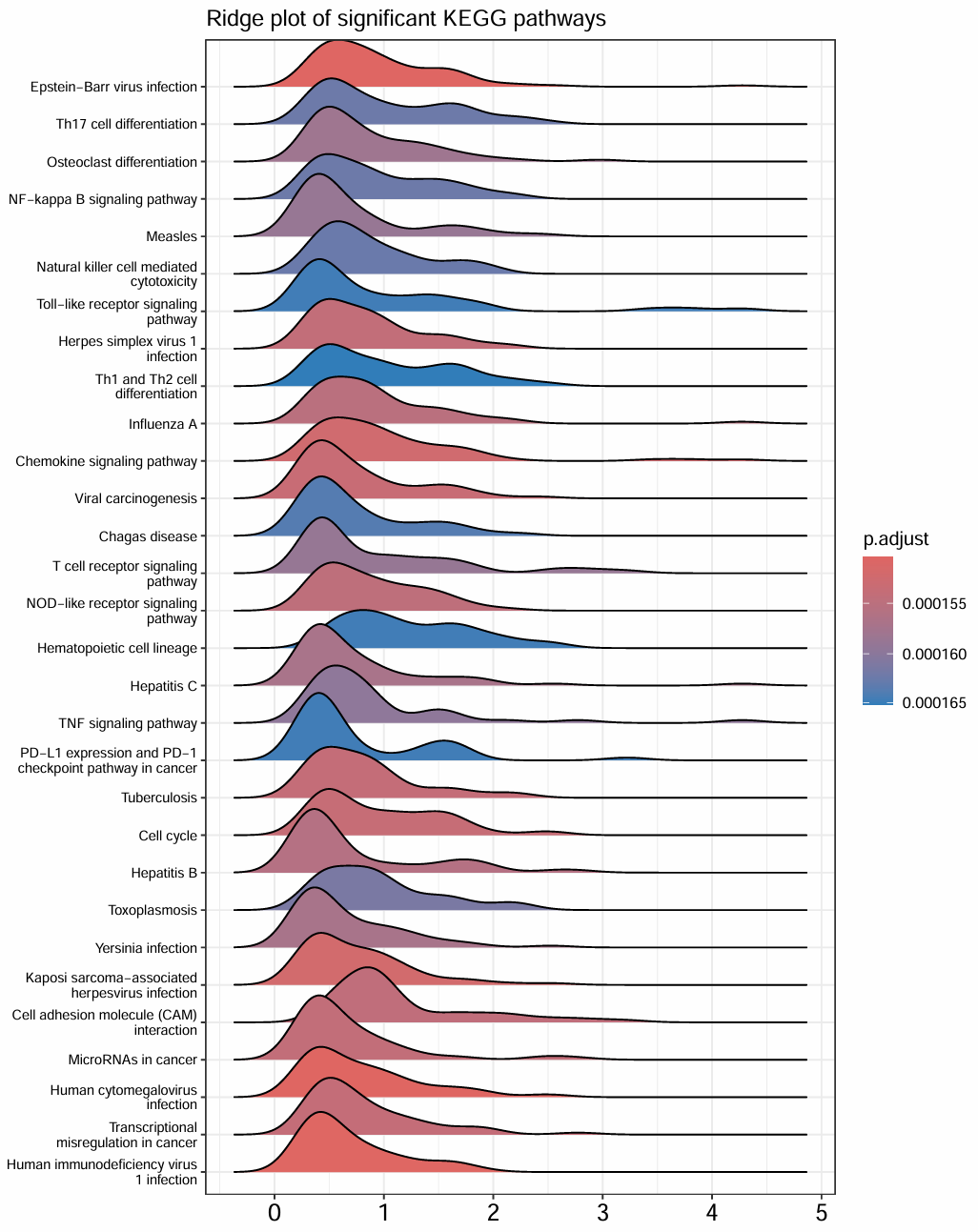

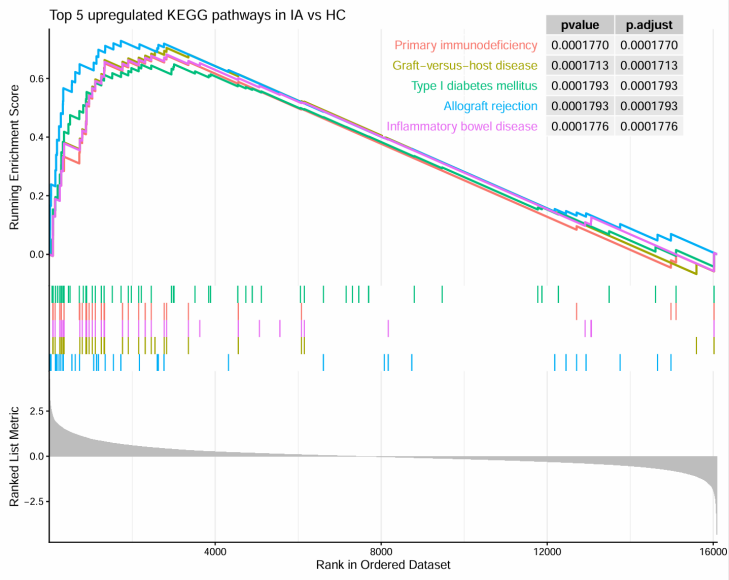


A

C

B


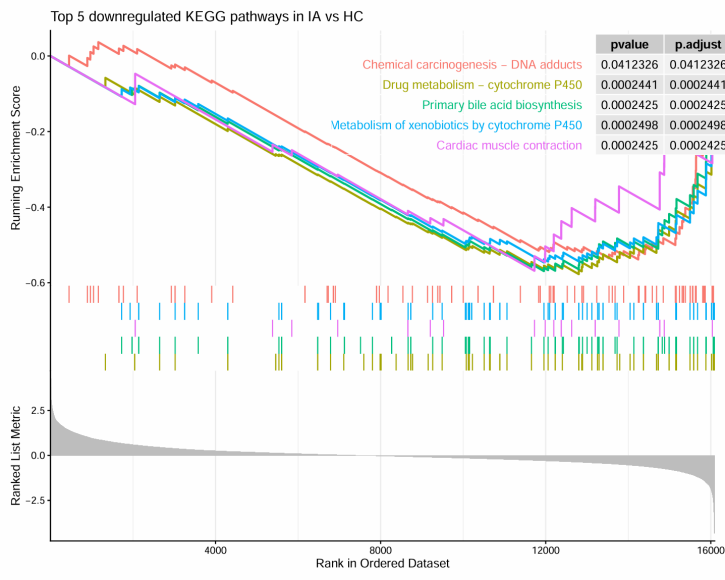


**Figure S2** | Gene set enrichment analysis (GSEA) of IA versus HC

A: Top 5 upregulated KEGG pathways in IA phase (with NES and *P*-values).

B: Top 5 downregulated KEGG pathways in IA phase (with NES and *P*-values).

C: Ridge plot showing distribution of all significant pathways.


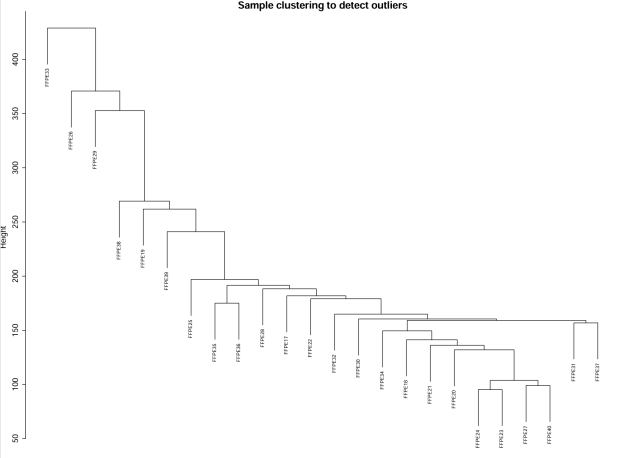

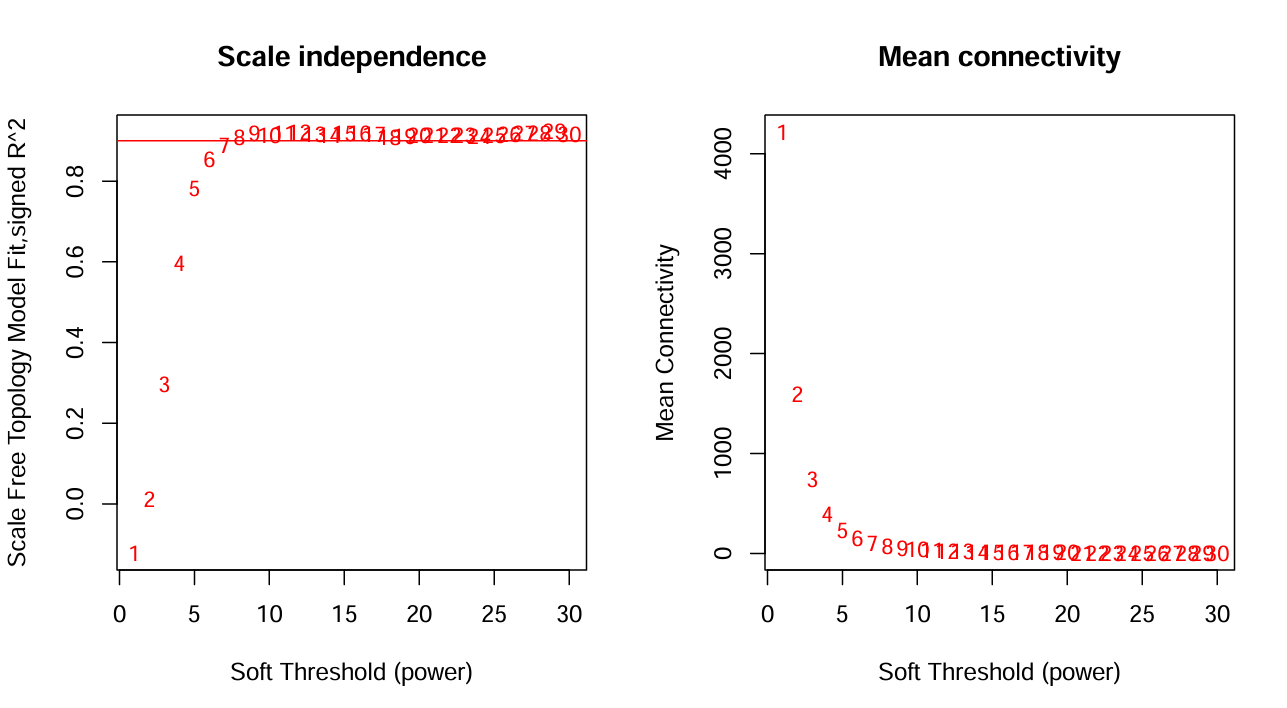


B

A

C


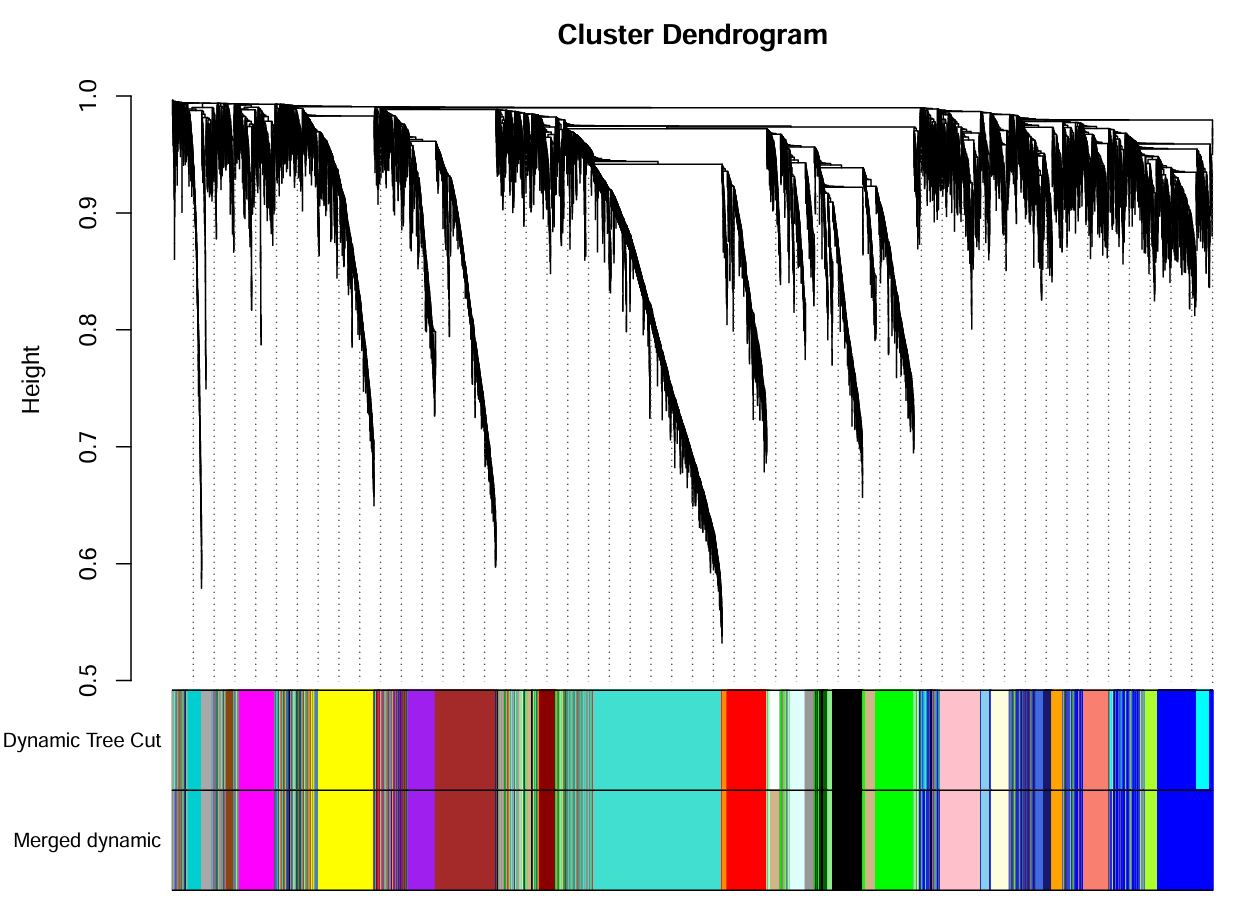

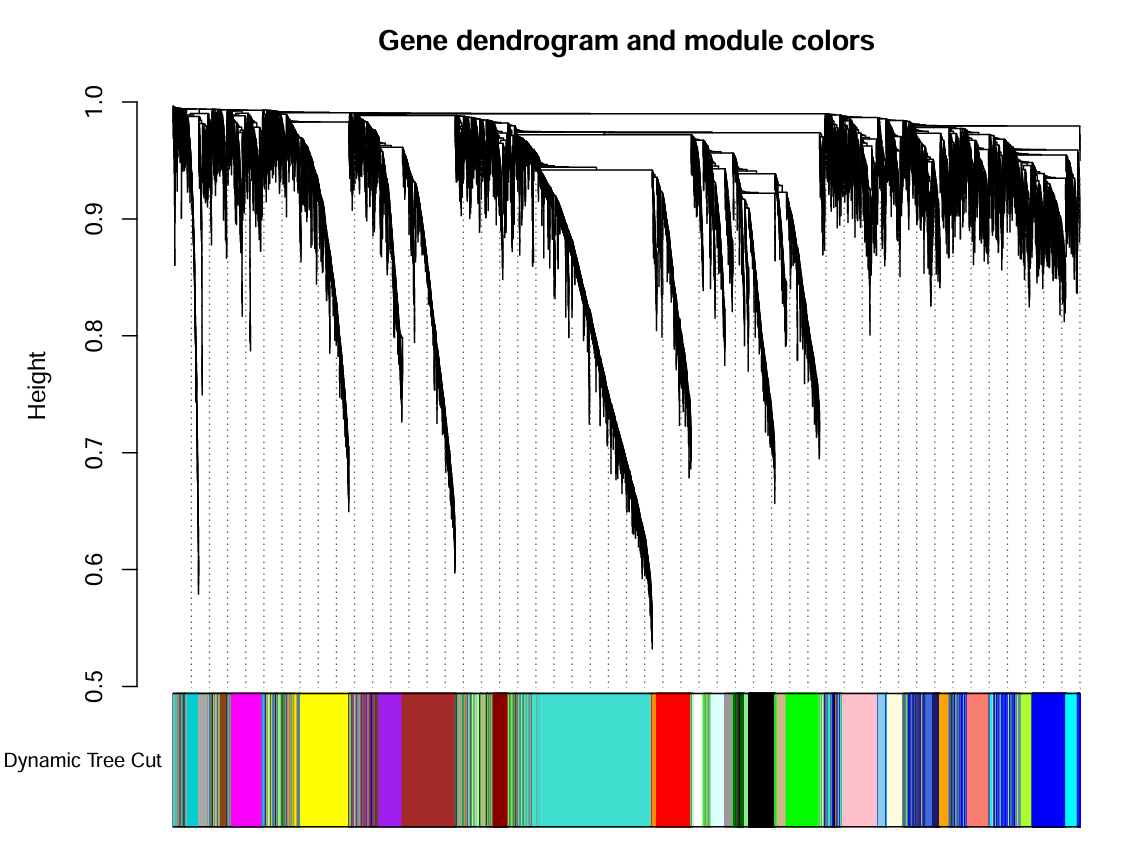


D

**Figure S3** | Weighted gene co-expression network analysis (WGCNA)

A: Determination of soft-thresholding power (scale-free fit index and mean connectivity).

B: Sample clustering dendrogram for WGCNA (with outlier removal).

C: Gene dendrogram and module colors (initial modules).

D: Merged module dendrogram after merging similar modules.


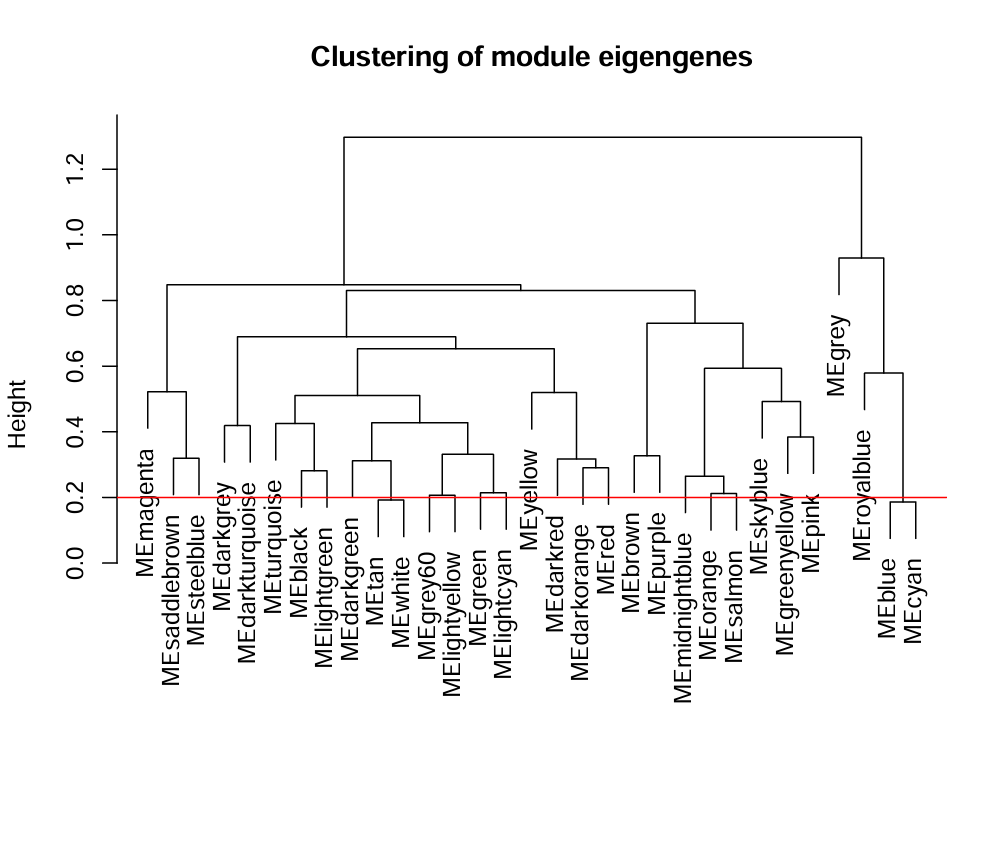

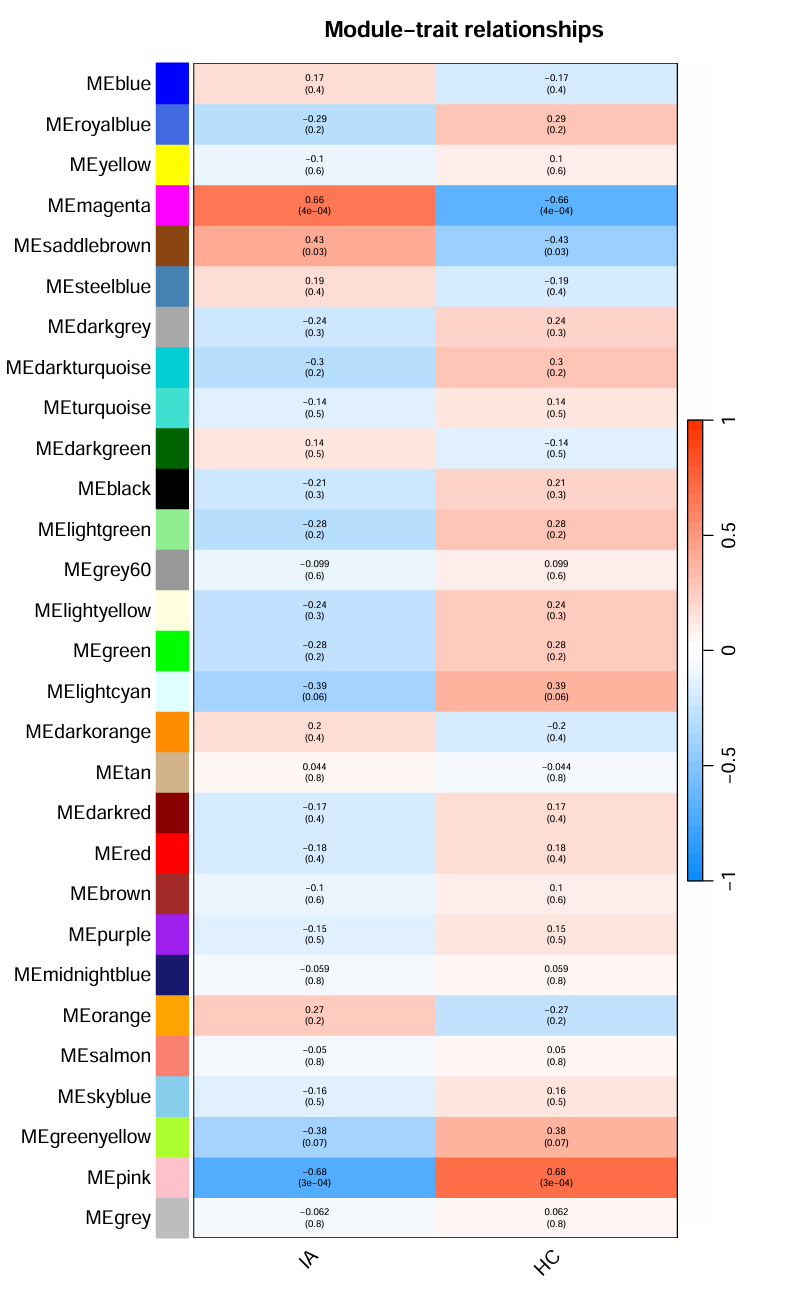


B

A


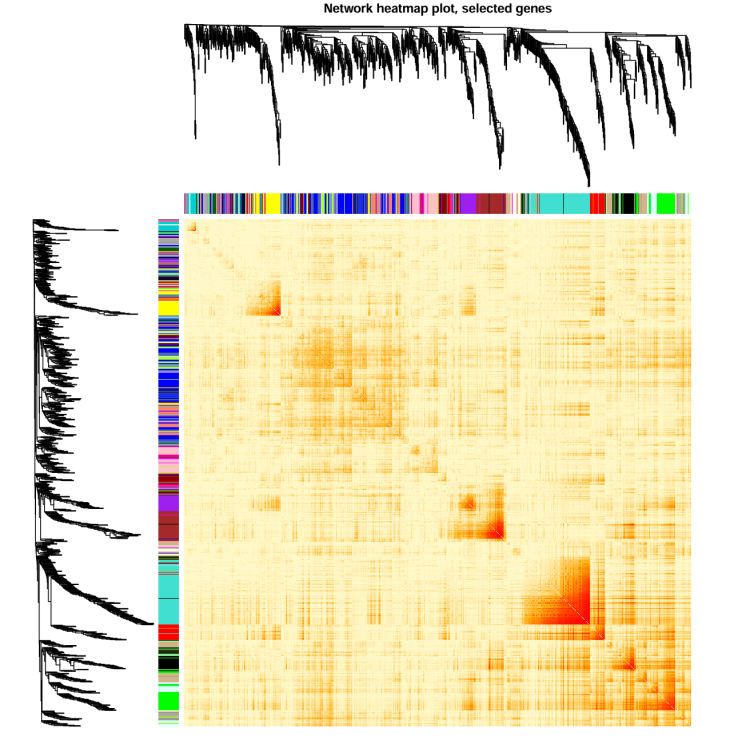


C

**Figure S4** | Characterization of WGCNA modules associated with IA phase

A: Heatmap of module-trait correlations.

B: Clustering dendrogram of module eigengenes.

C: Network heatmap plot of selected modules.

B

A


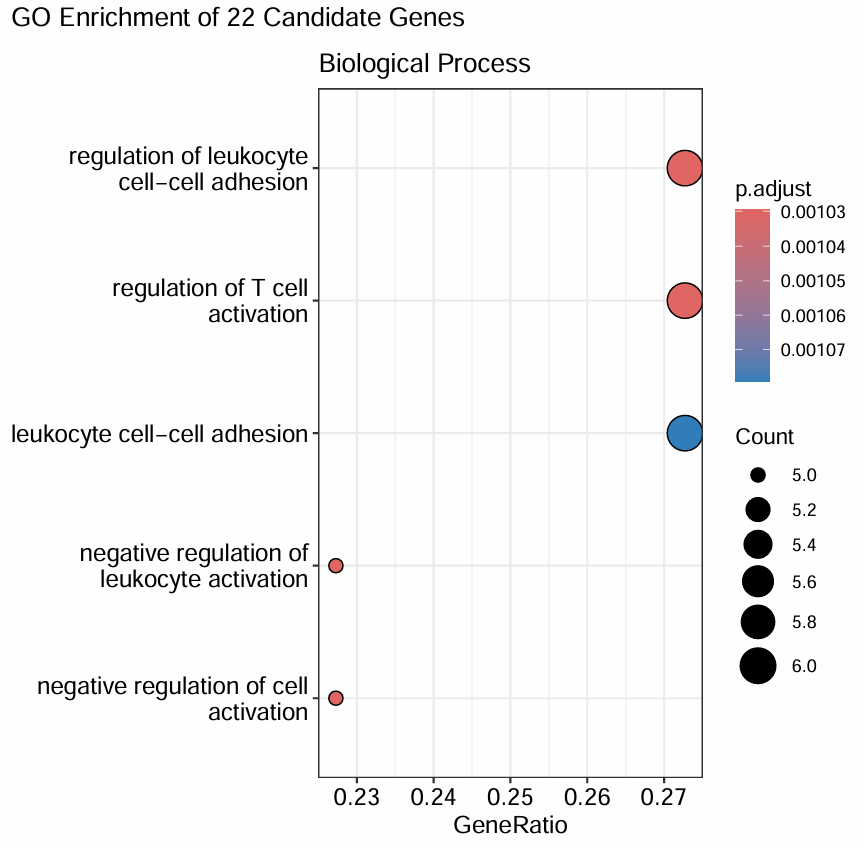

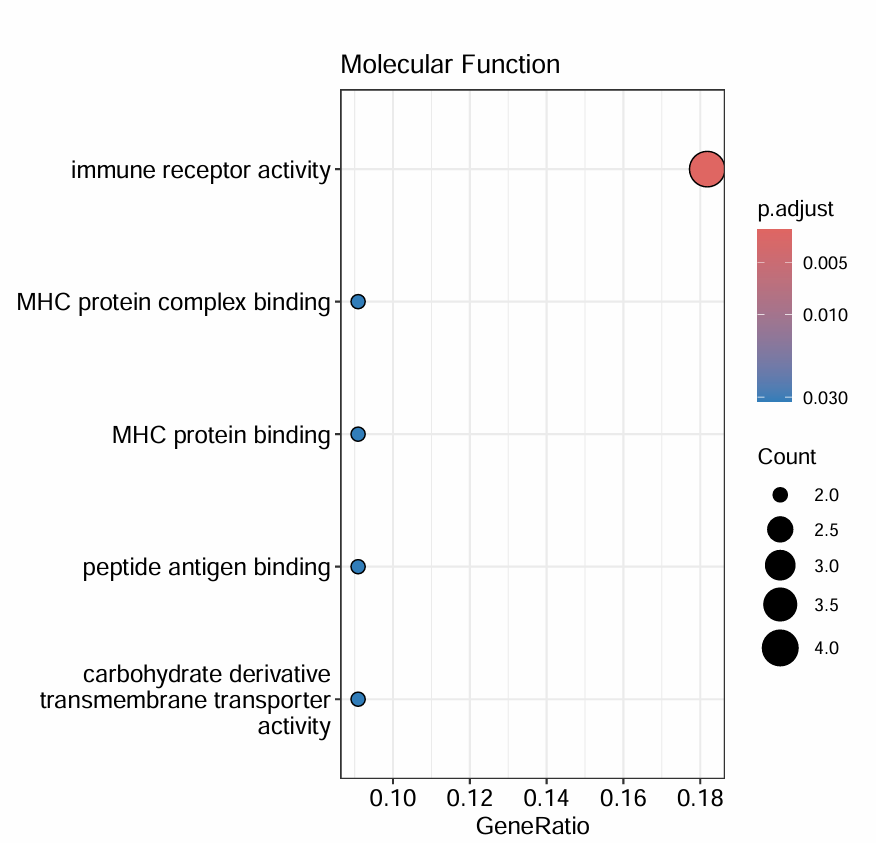

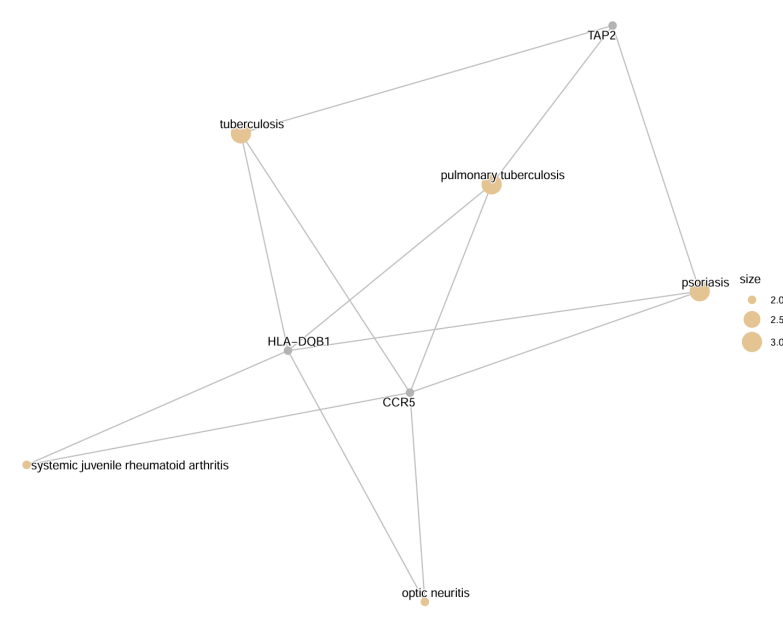

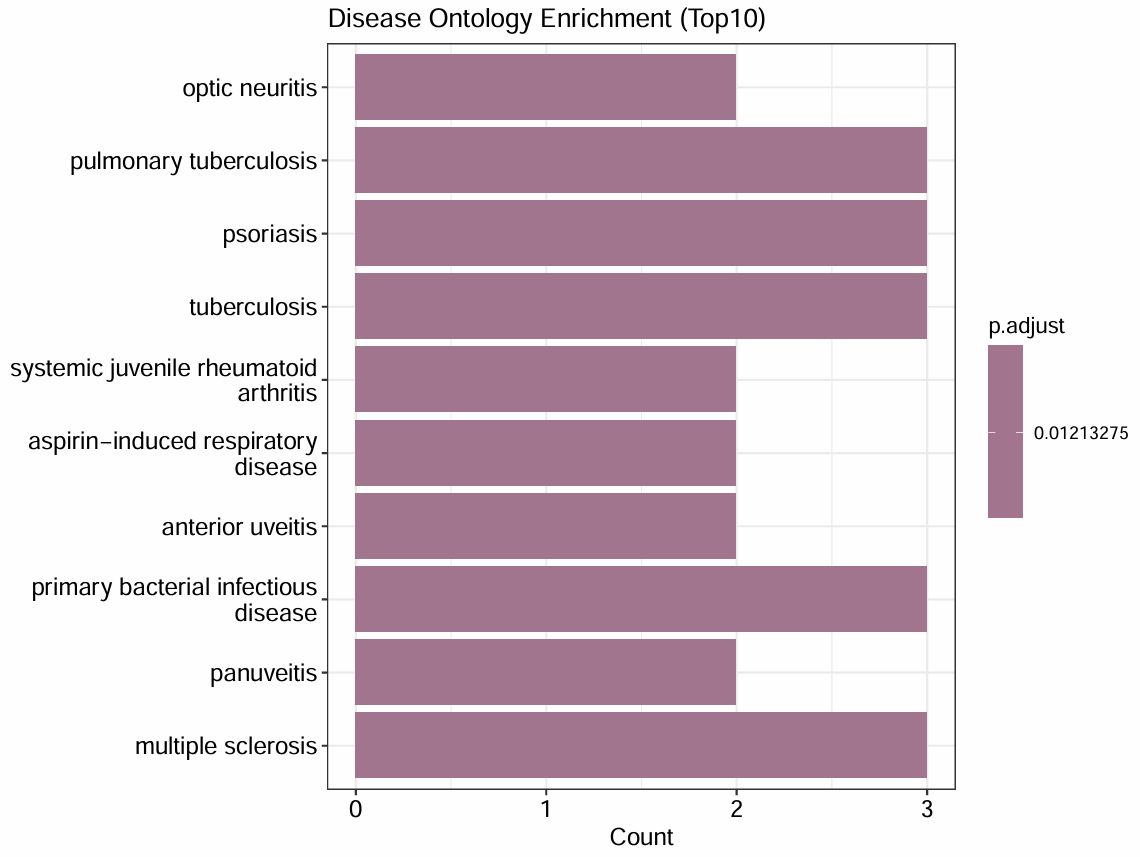


C

D

**Figure S5** | Functional enrichment analysis of 22 candidate genes

A-B: Disease Ontology (DO) enrichment (bar plot and network plot).

C-D: Gene Ontology (GO) enrichment (bubble plots for BP, MF).


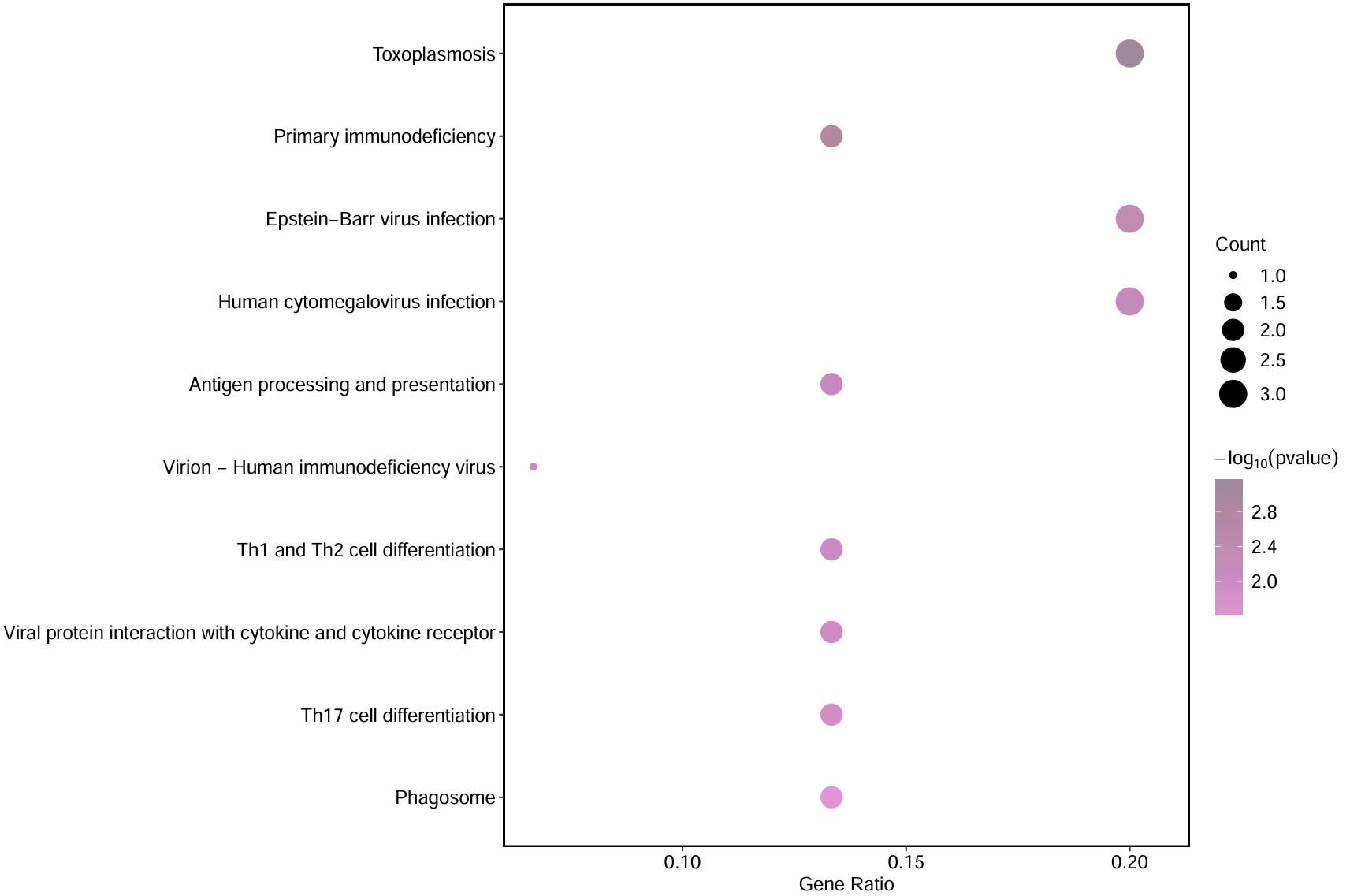


B

A


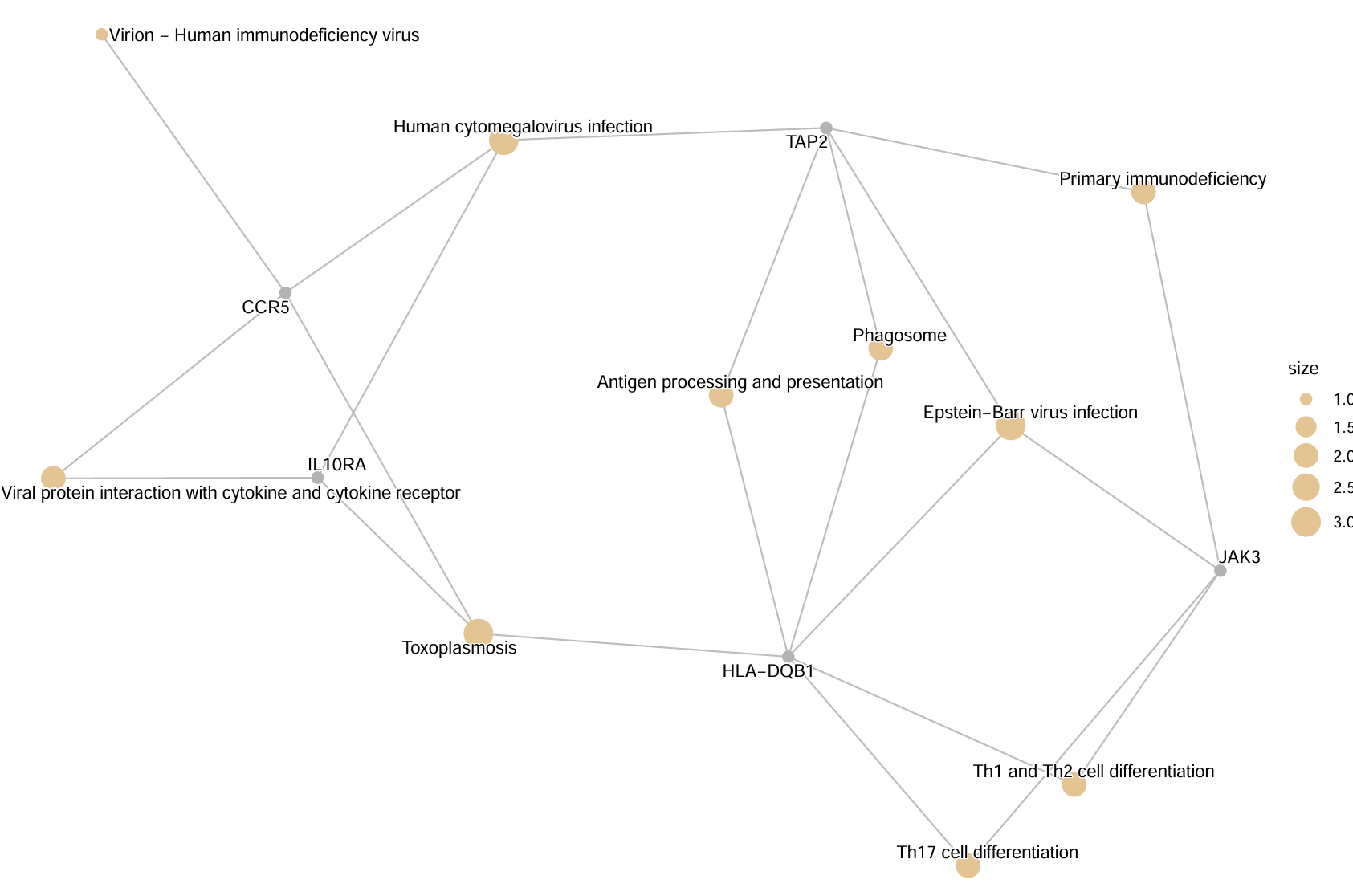


**Figure S6** | KEGG pathway enrichment of 22 candidate genes

A: Bubble plot of top enriched KEGG pathways.

B: Network plot showing gene-pathway relationships.


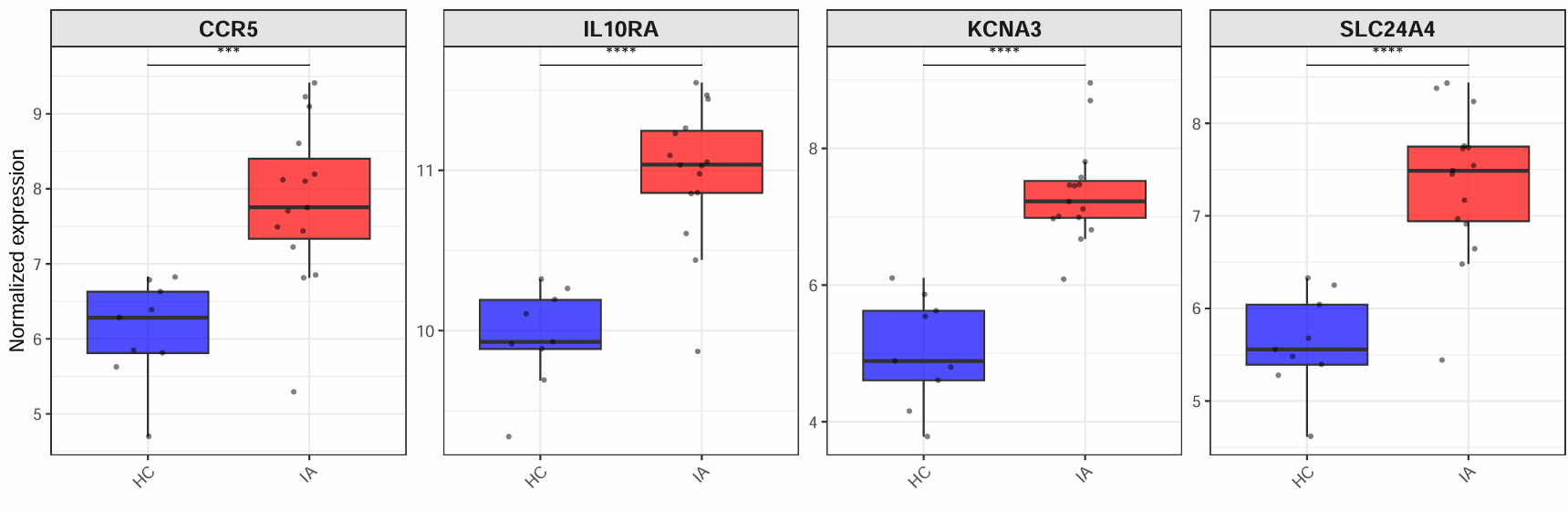


D

C

B

A

**KCNA3**

**CCR5**

F

E


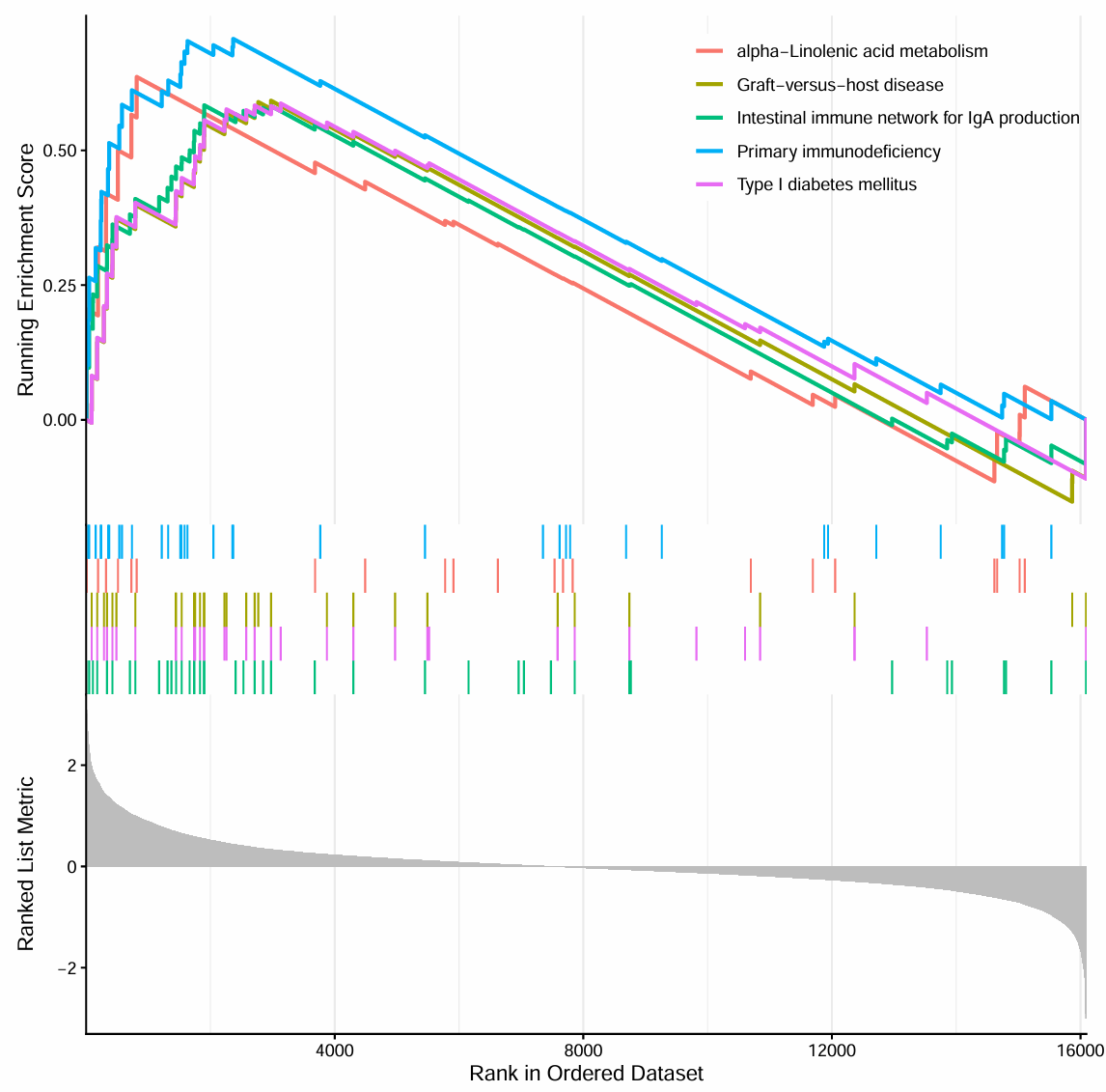

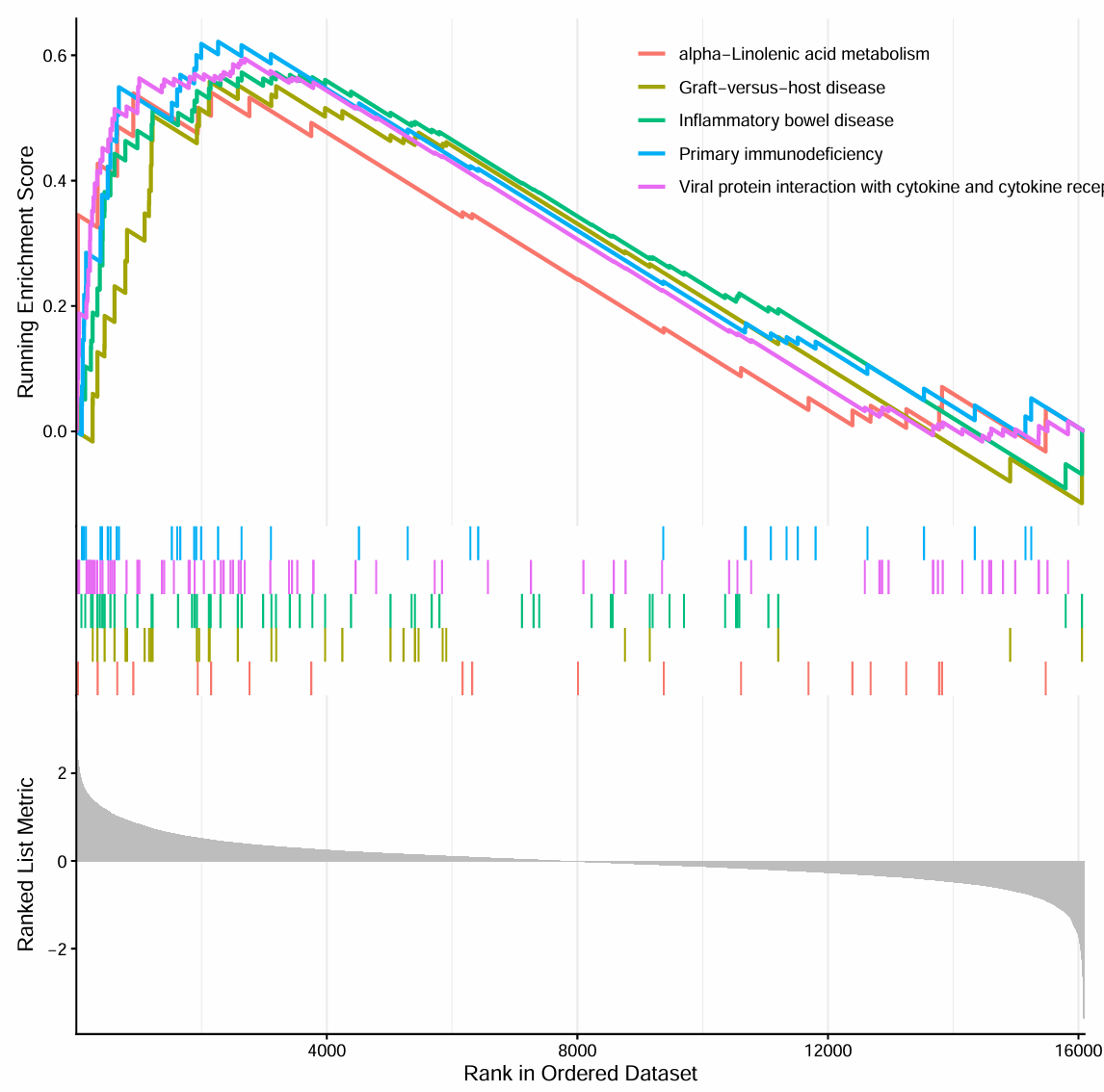


H

G

**SLC24A4**

**IL10RA**


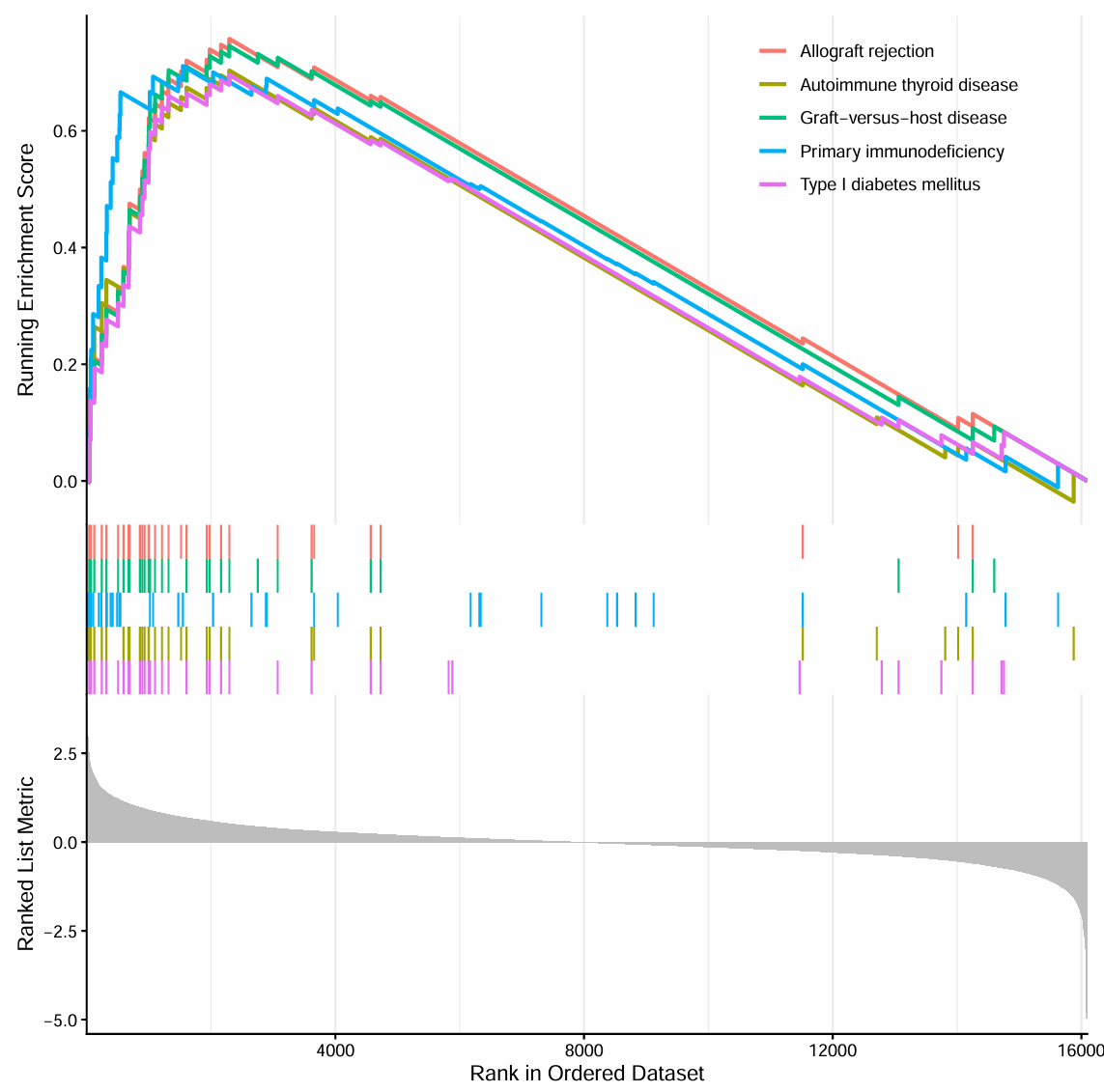

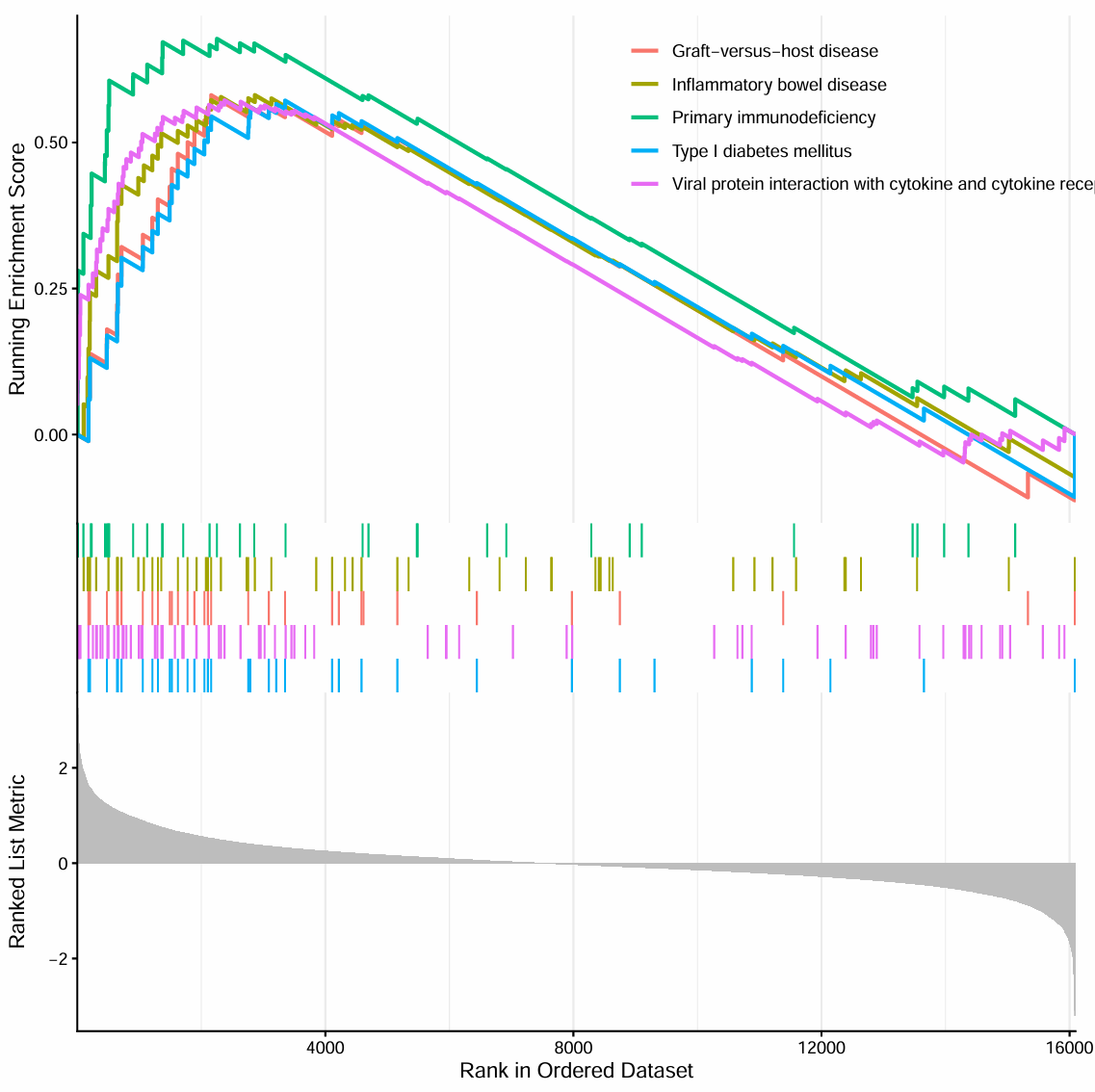


**Figure S7** | Expression and analysis of hub genes in GSE230397

A-D: Box plots of CCR5, KCNA3, SLC24A4, IL10RA expression in IA vs HC (****P*<0.001,*****P*<0.0001, XXXX test).

E-H: GSEA enrichment plots for each hub gene high expression group (top 5 pathways).


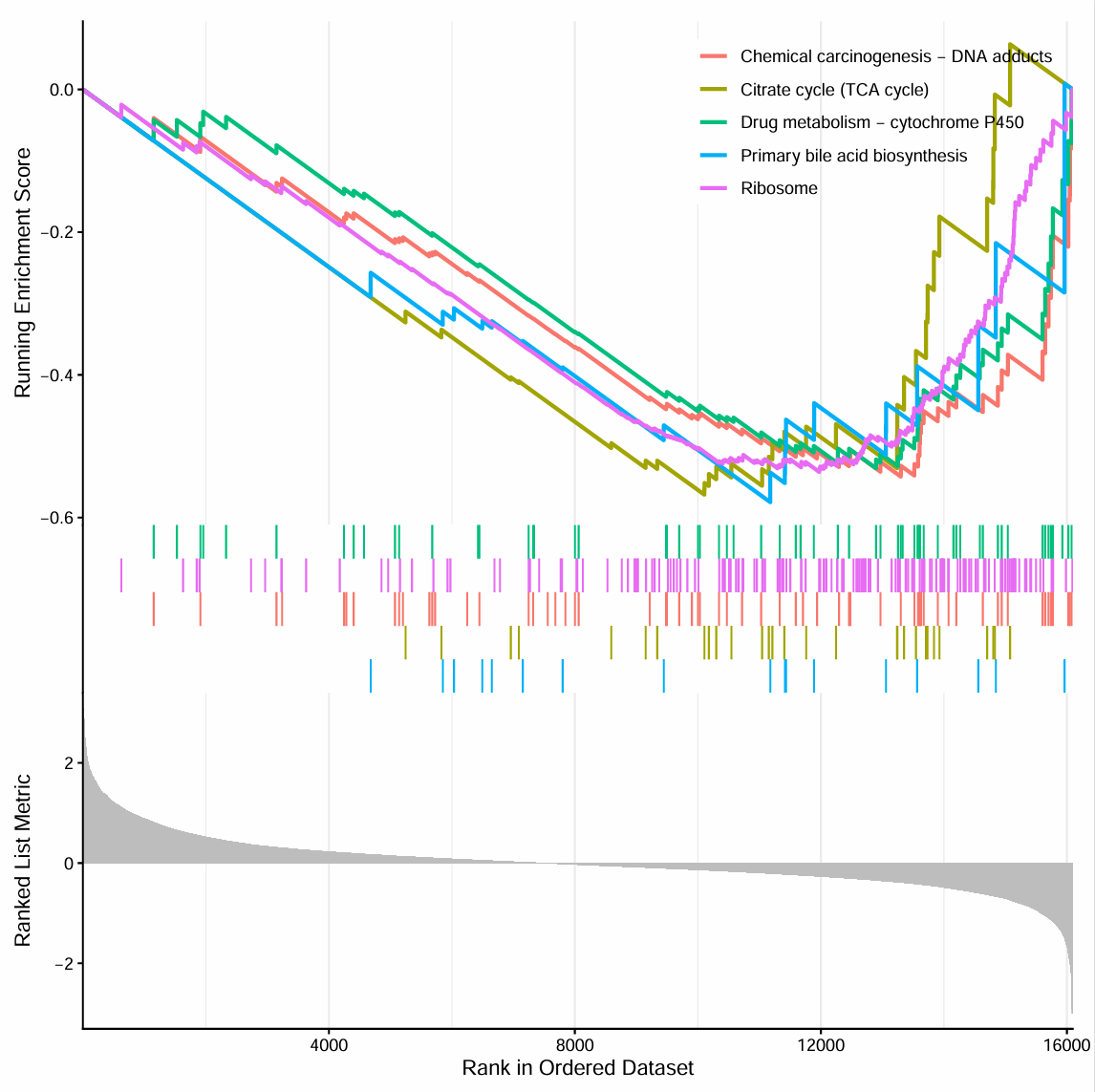

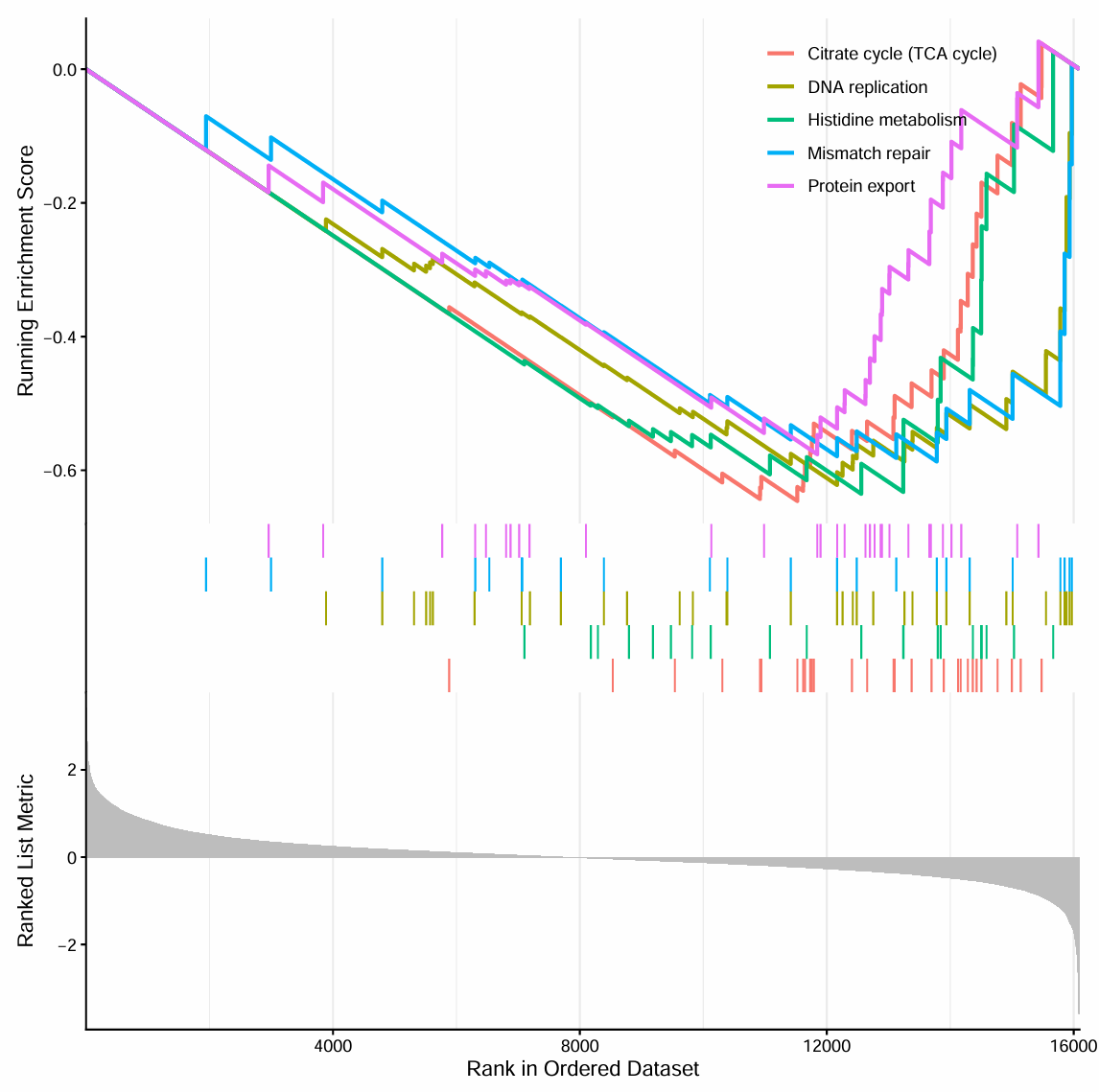


**CCR5**

**KCNA3**

A

B

C

D


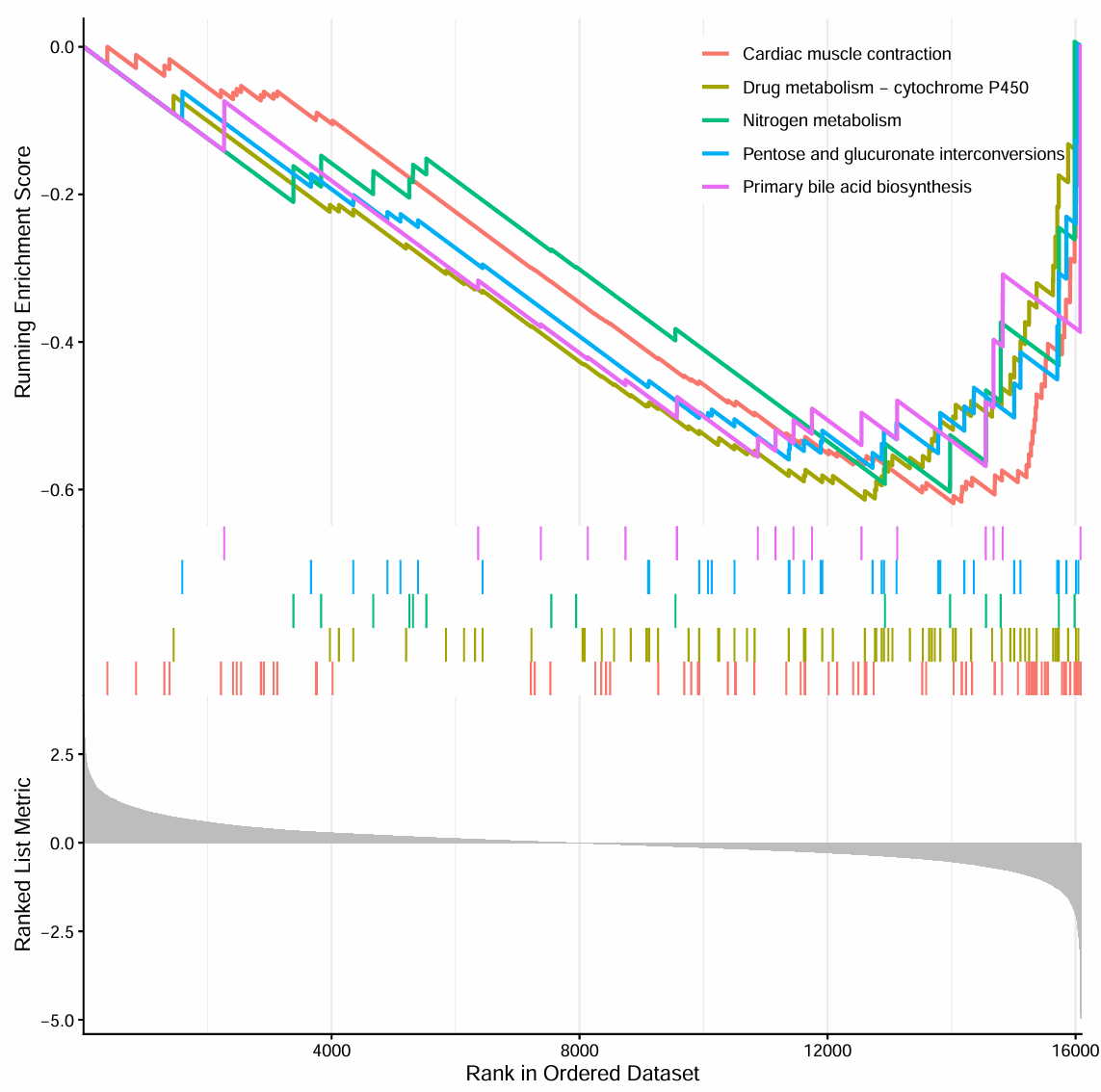

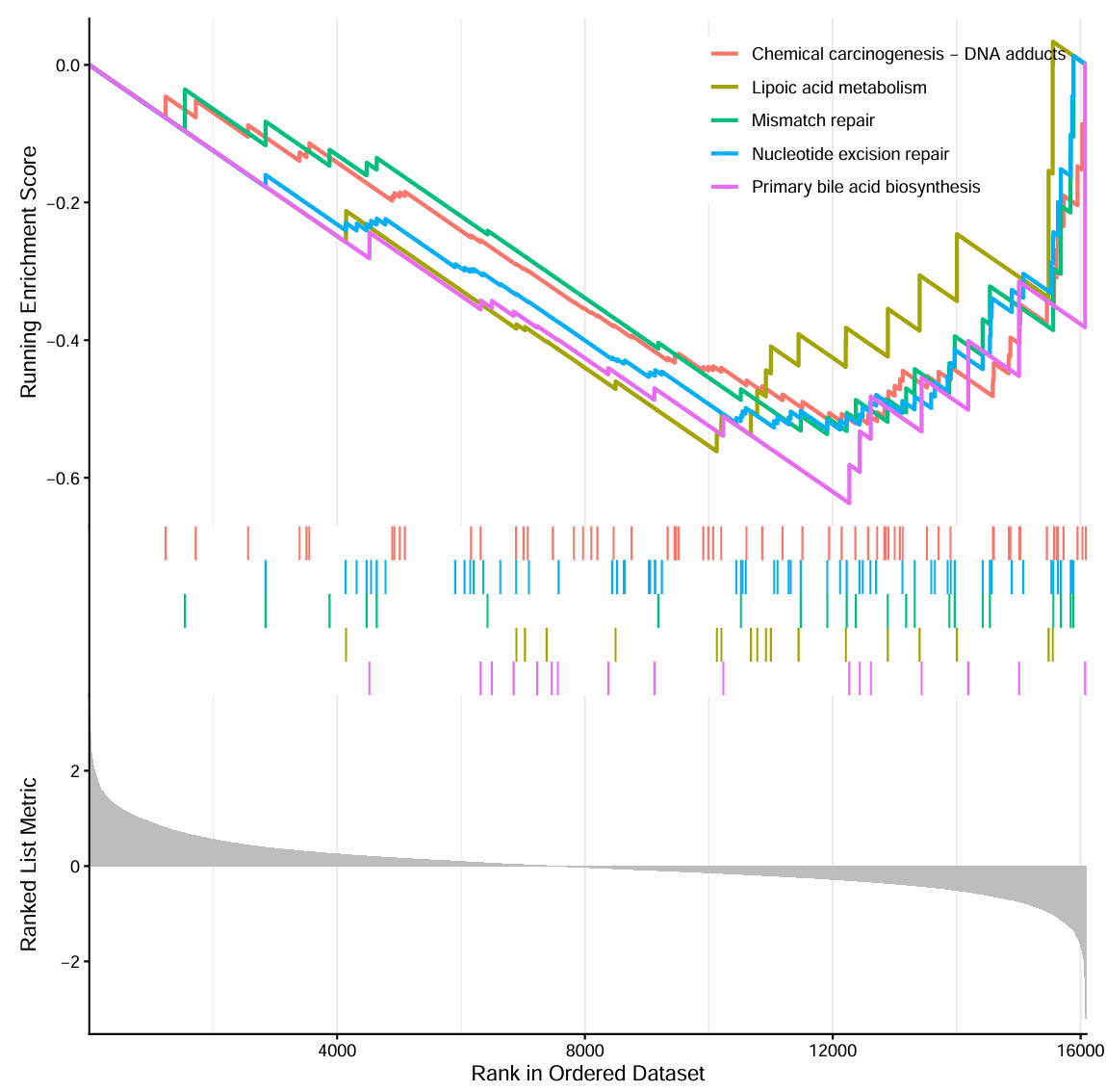


**IL10RA**

**SLC24A4**

**Figure S8** | GSEA for low expression groups of hub genes

A-D: GSEA plots for CCR5, KCNA3, SLC24A4, IL10RA low expression groups (top 5 pathways).


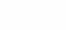

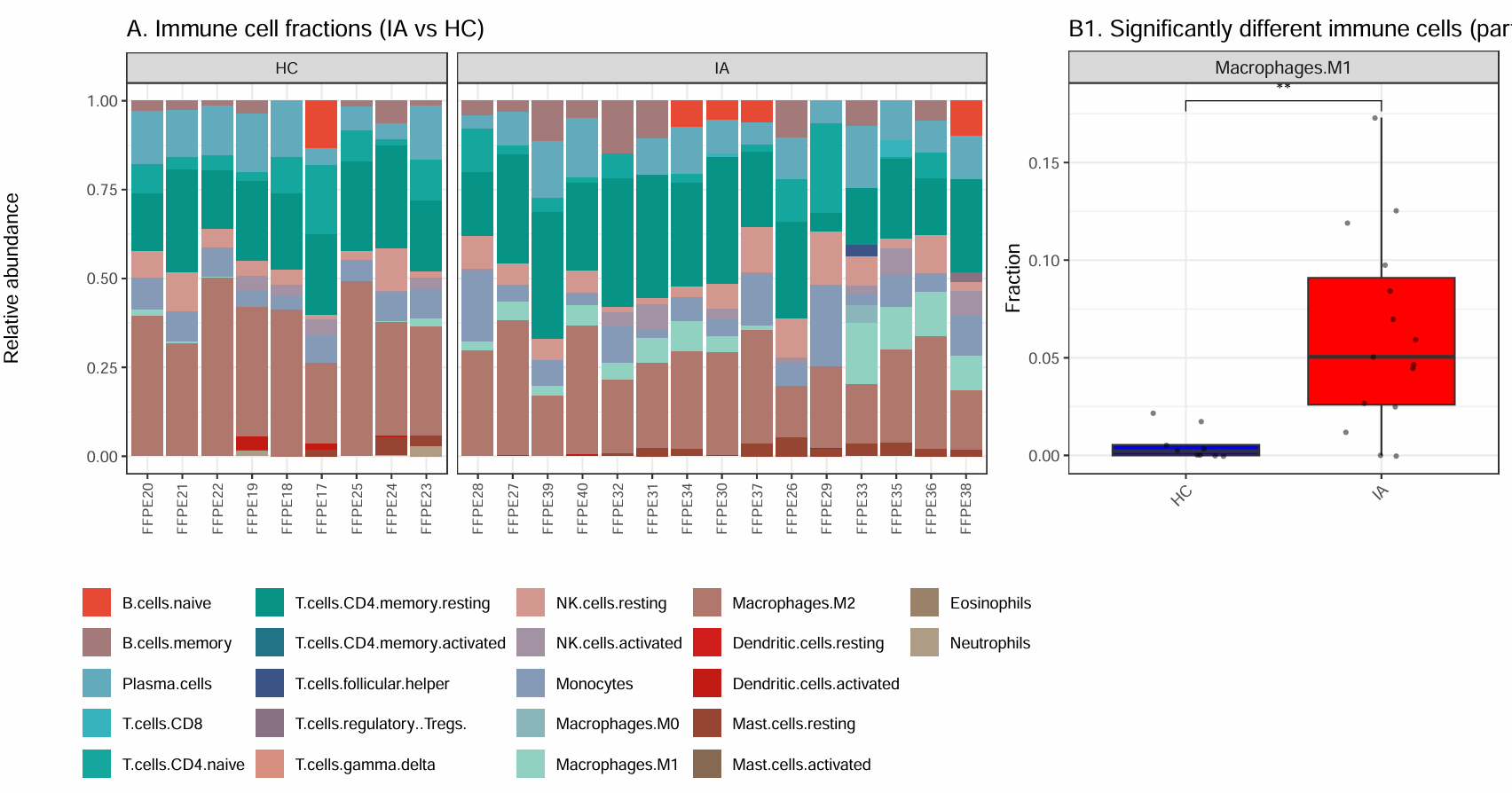


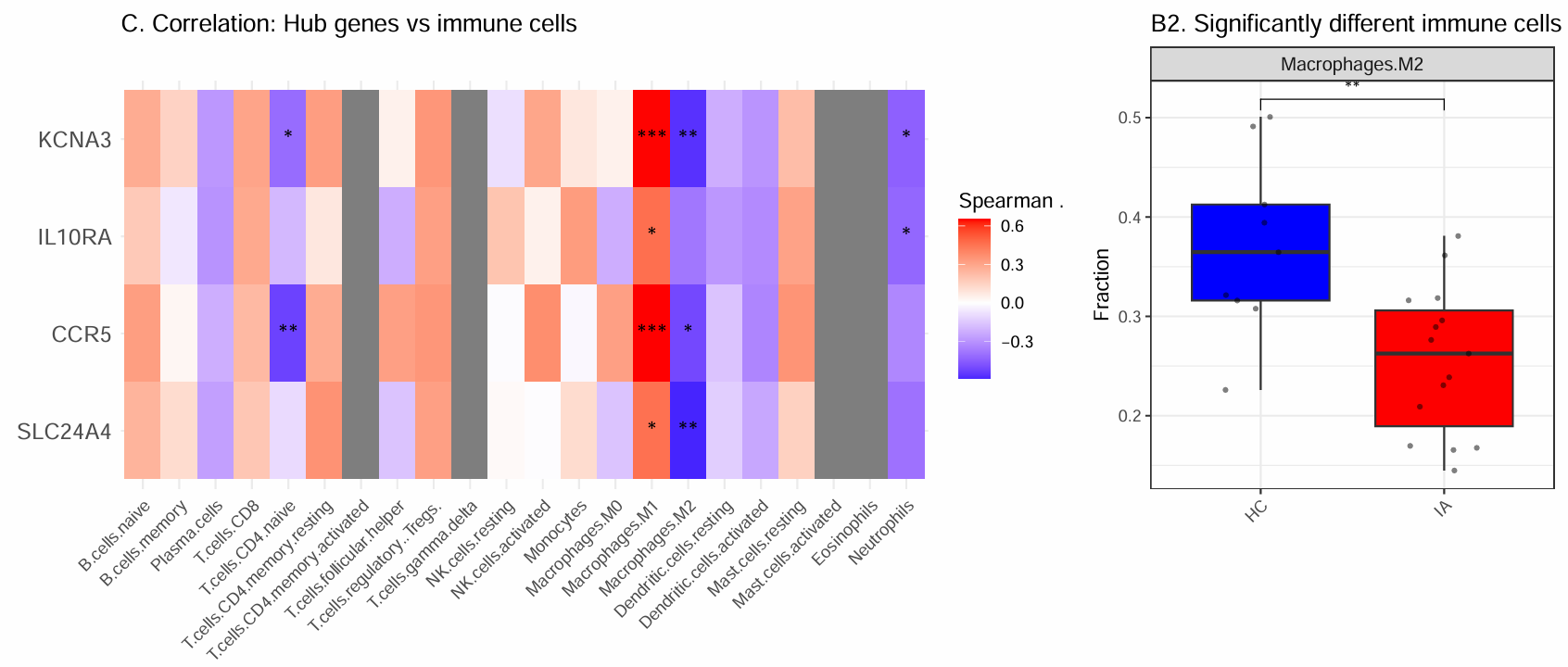


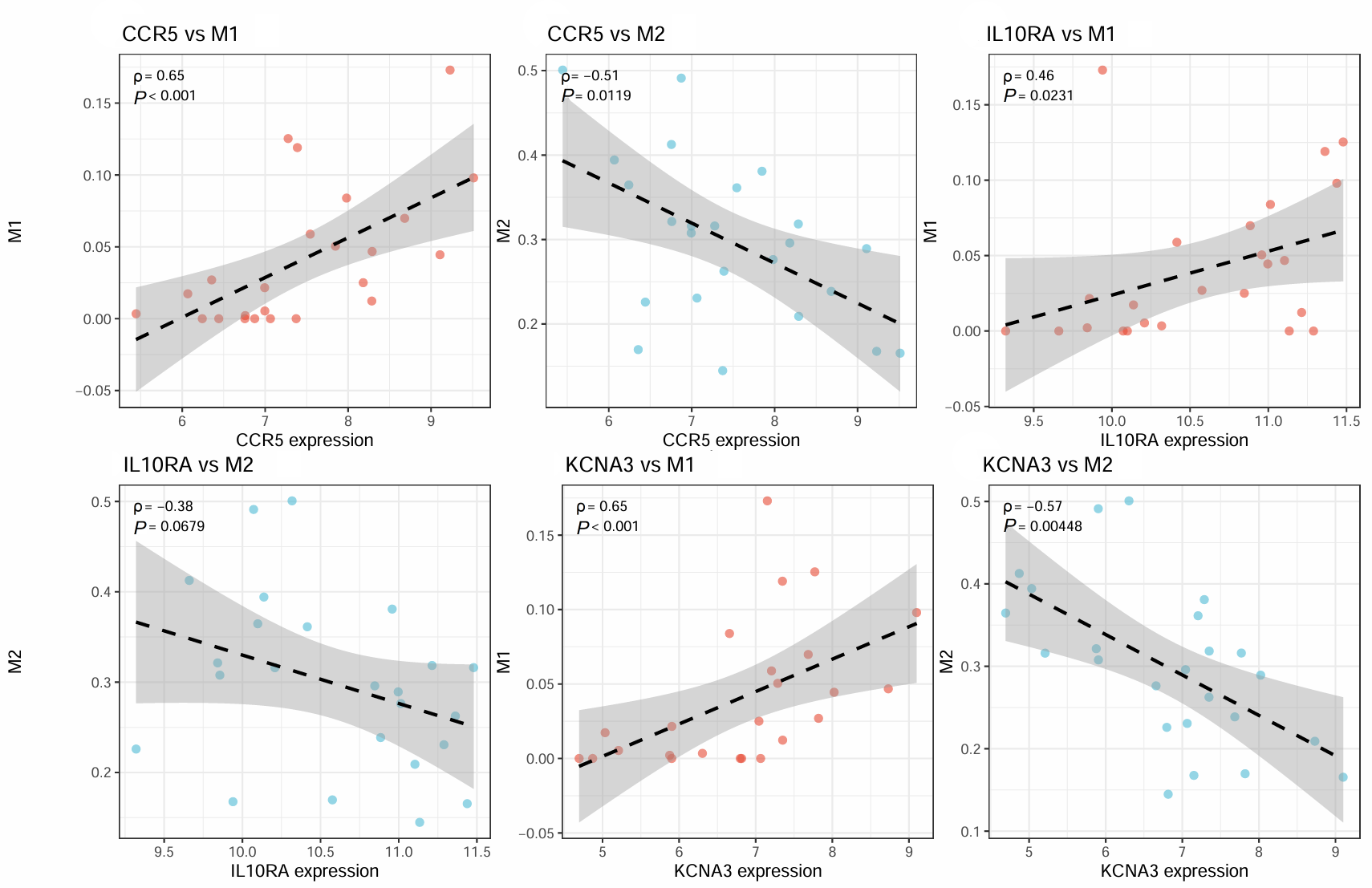


D.

**Figure S9** | Immune infiltration analysis of liver tissue

A: Stacked bar plot of 22 immune cell fractions in IA and HC.

B: Box plots comparing IA and HC groups for immune cell types that showed significant differences. Statistical annotations are included. Subpanels: B1, Macrophages M1; B2, Macrophages M2 (***P*<0.01, XXXX test).

C: Correlation heatmap between hub genes and immune cell fractions (complete 22 cell types) (**P*<0.05, ***P*<0.01, ****P*<0.001, XXXX test).

D: Scatter plots of CCR5, IL10RA, and KCNA3 correlation with the infiltration proportion of M1 and M2 macrophages (as SLC24A4 is shown in main Figure 3D).

A


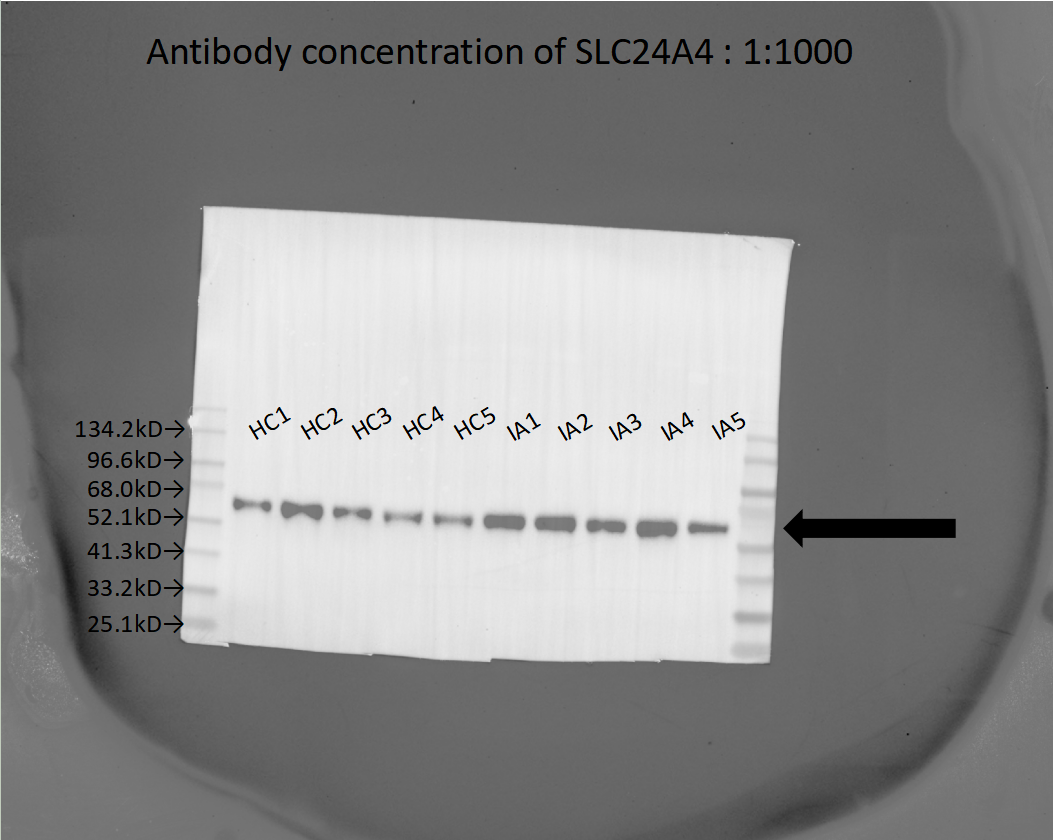


SLC24A4

B


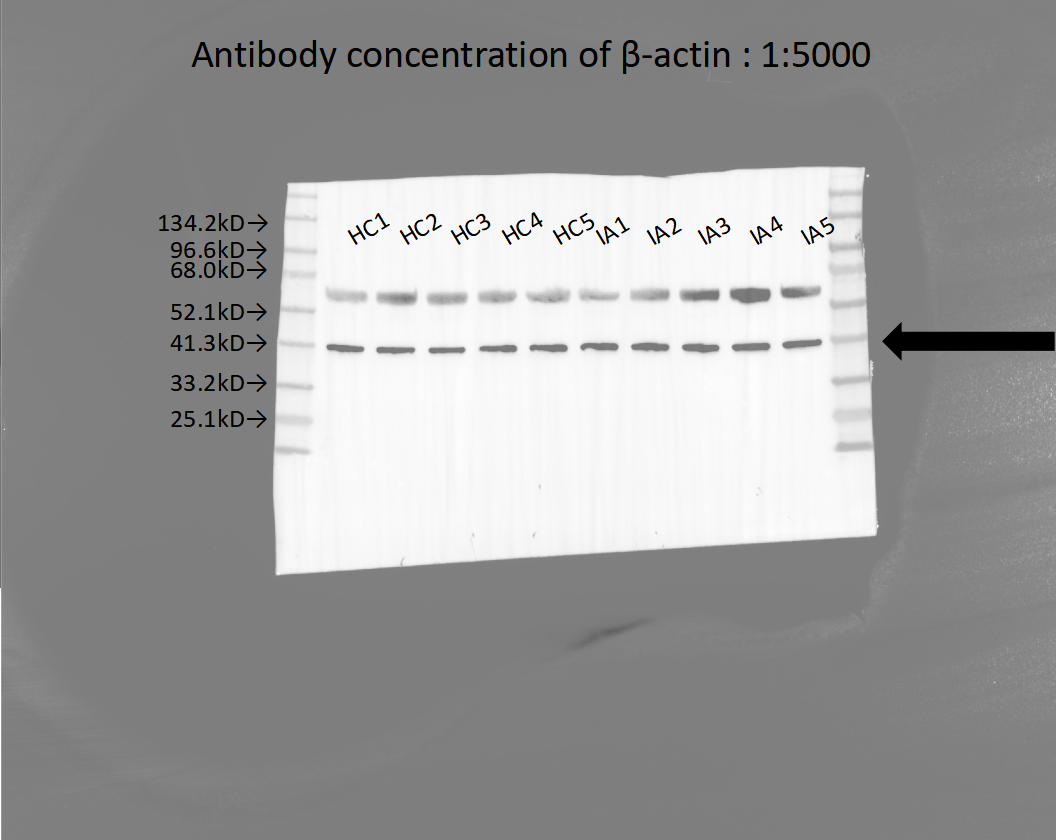


β-actin

**Figure S10** | Full Western blot images

A: Uncropped blots for SLC24A4 (5 IA + 5 HC).

B: Uncropped blots for β-actin (same samples).


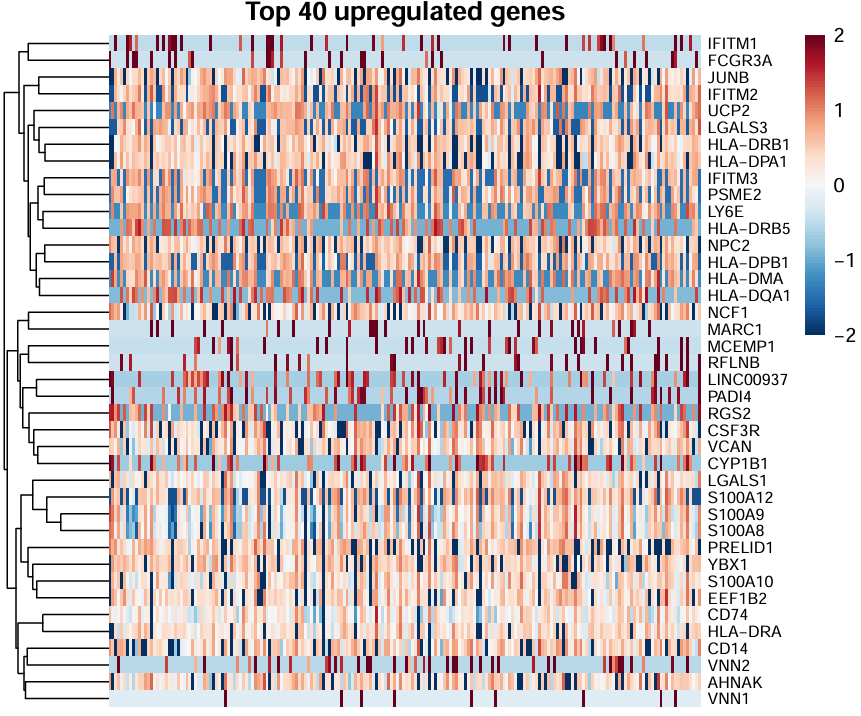


A

B


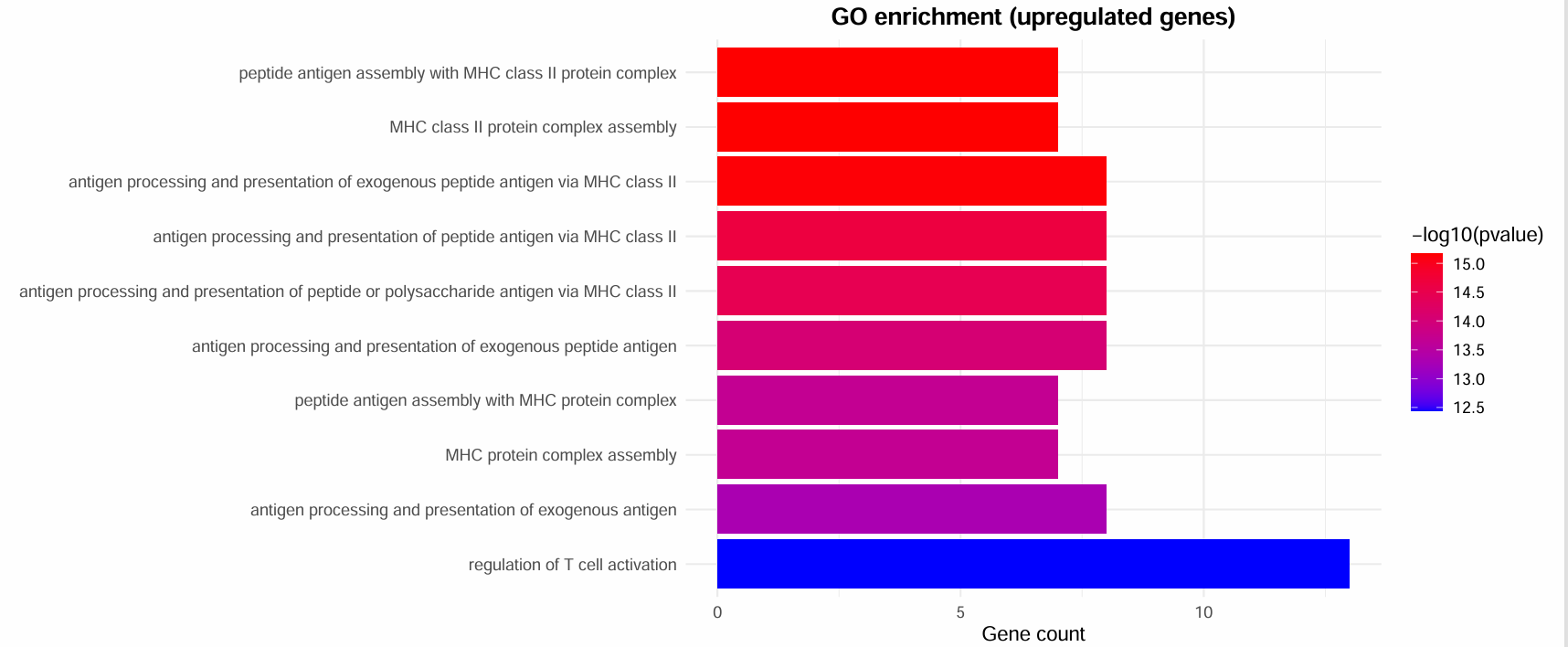


C


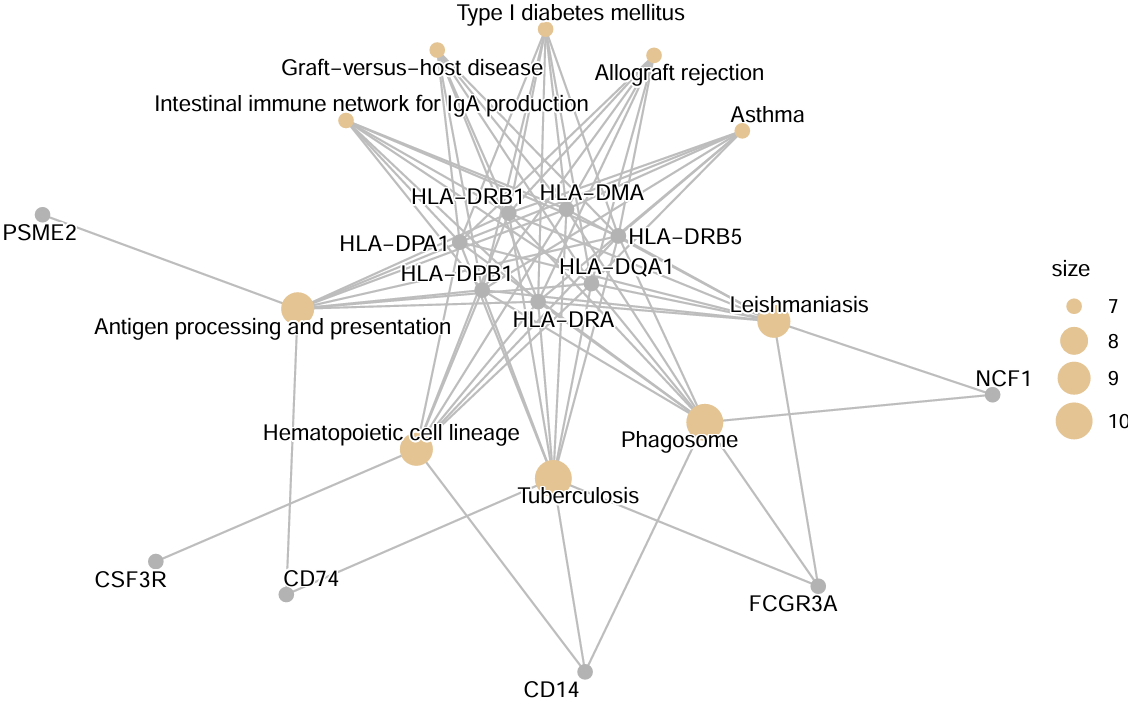


**Figure S11** | *In silico* knockout analysis of SLC24A4 in classical monocytes

A: Heatmap of top 40 upregulated genes after *In silico* knockout of SLC24A4.

B: GO enrichment bar plot of upregulated genes after *In silico* knockout of SLC24A4 (top 10 terms).

C: KEGG pathway enrichment network after *In silico* knockout of SLC24A4.


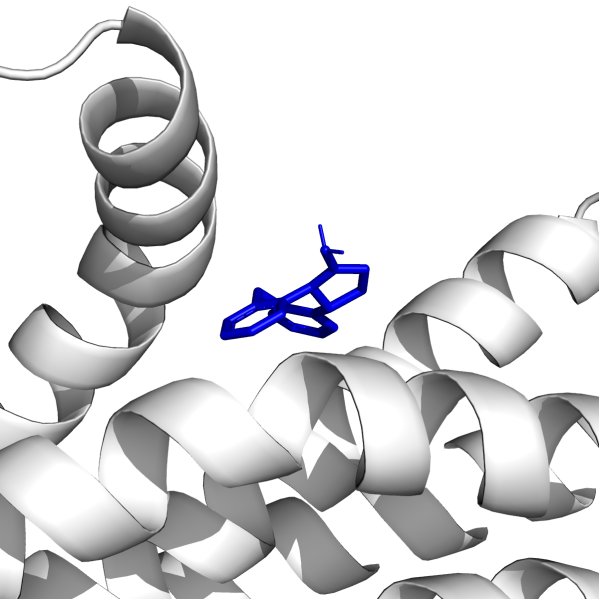

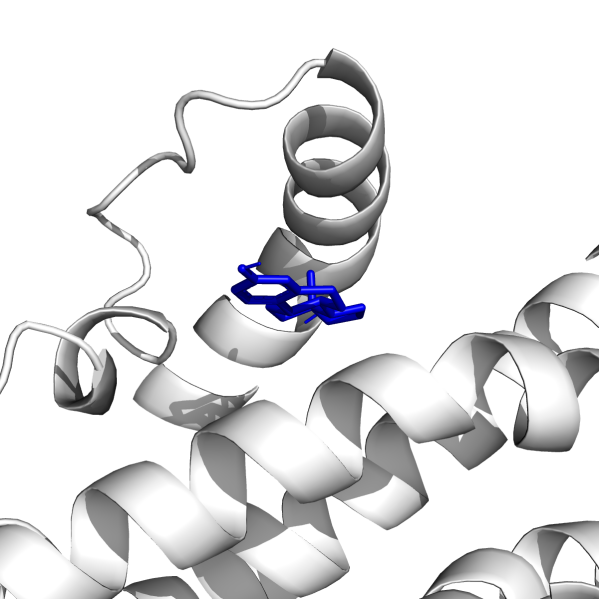

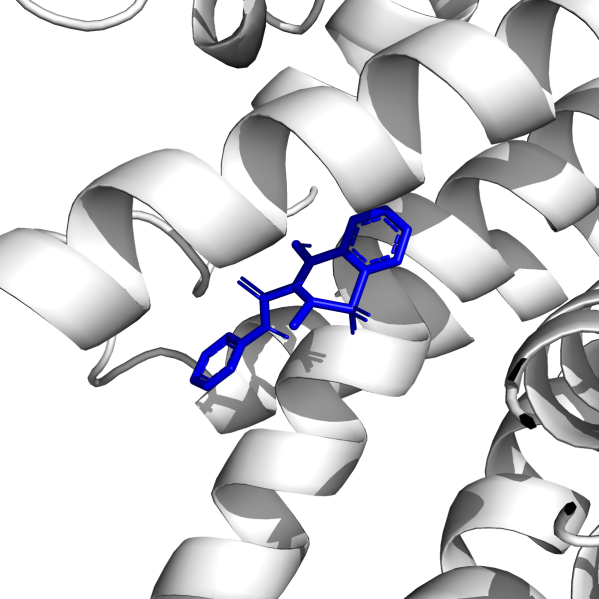

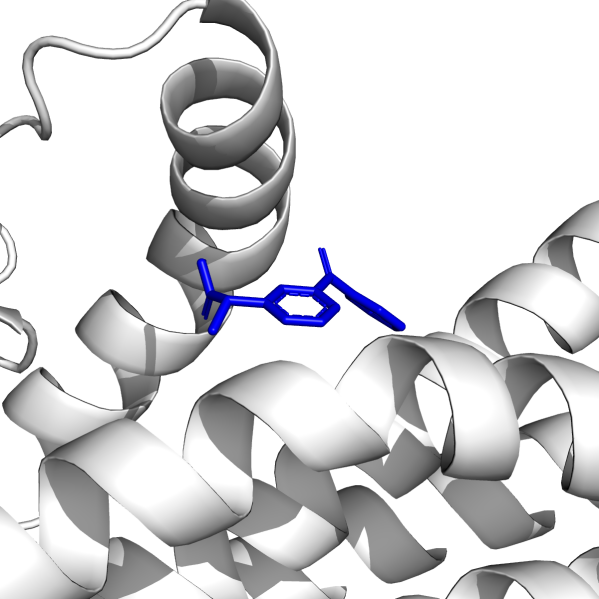

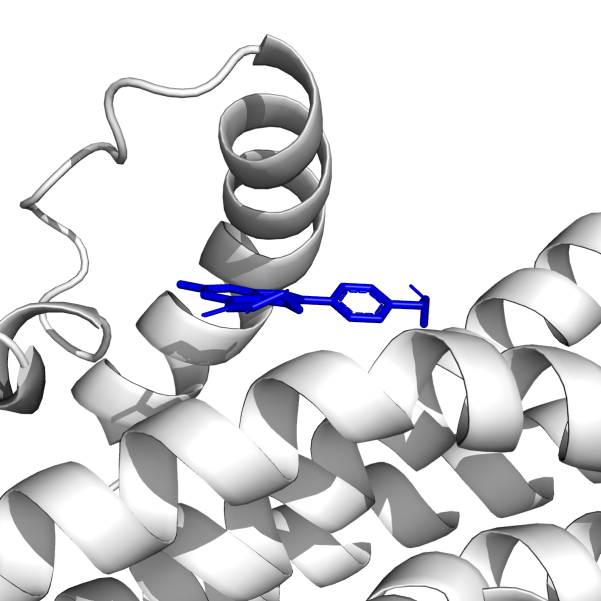

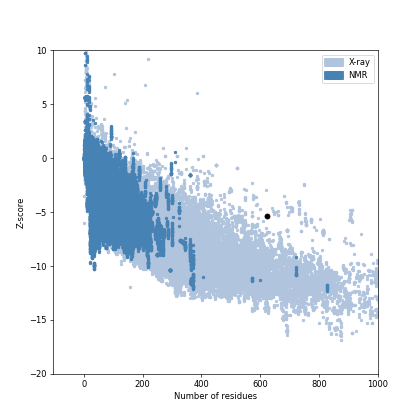


C

D

A

B

E

F

G

H


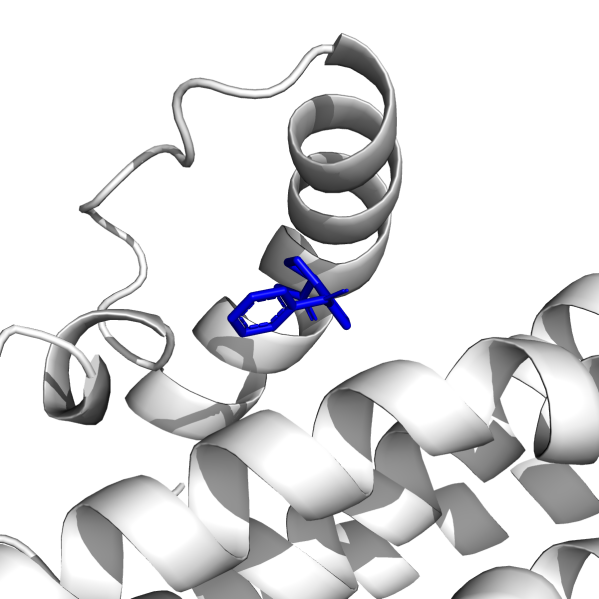

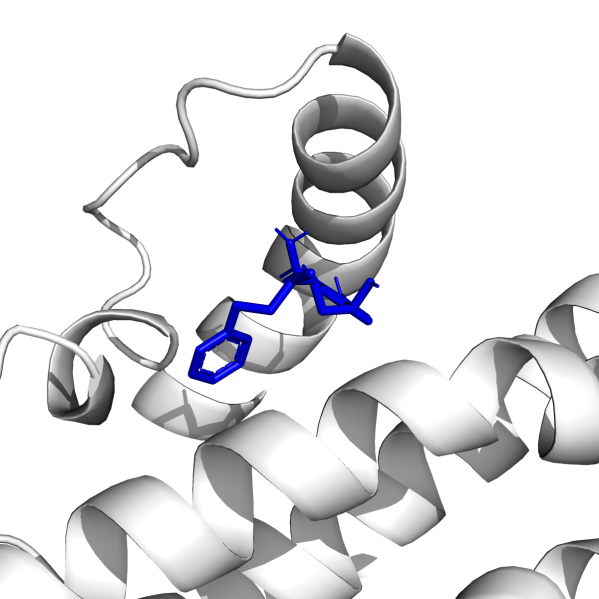


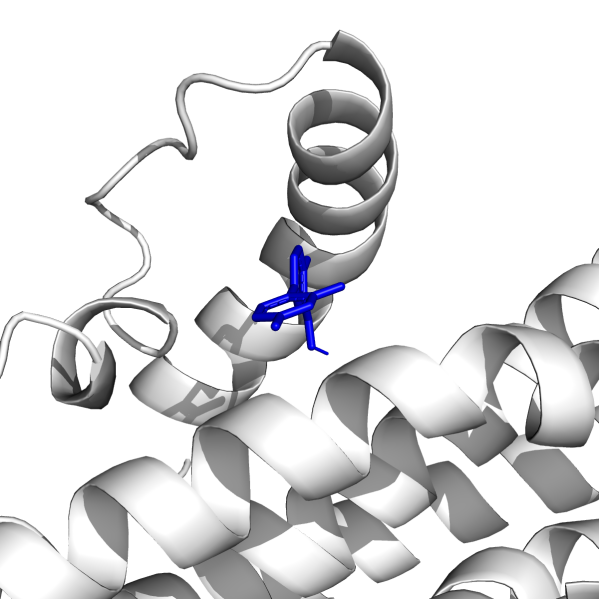

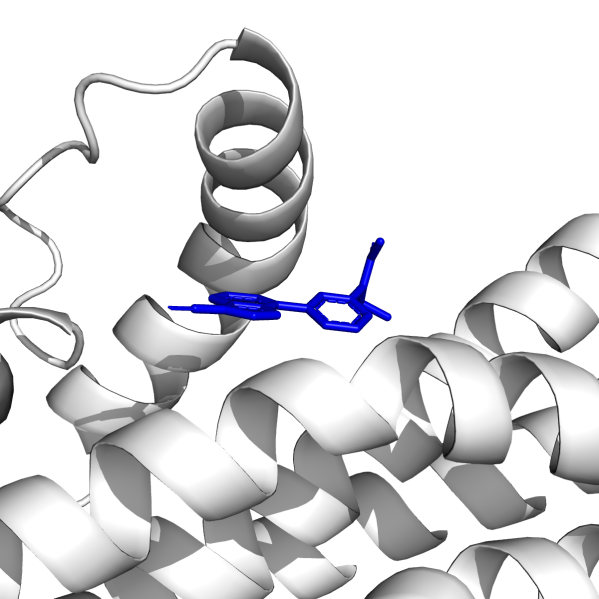

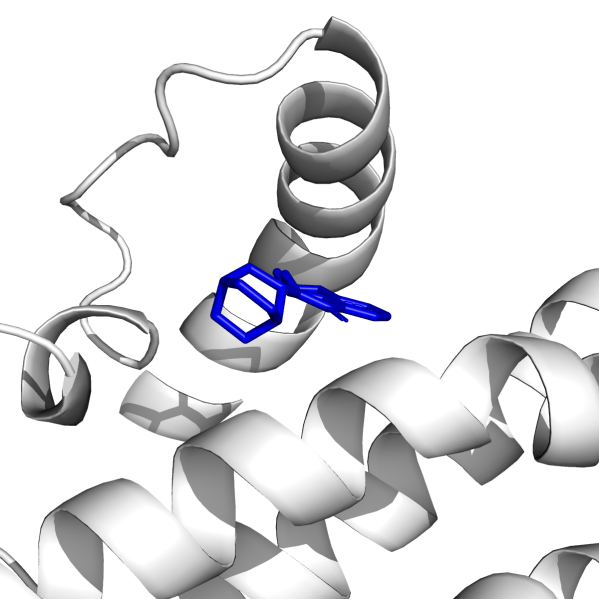


J

I

K

**Figure S12** | Structural validation and binding poses of top virtual screening hits targeting SLC24A4

A: ProSA-web Z-score plot assessing the overall quality of the AlphaFold-predicted SLC24A4 structure. The Z-score fell within the range characteristic of native protein structures of similar size.

B-K: Predicted binding poses of the top 10 compounds from molecular docking. SLC24A4 is shown as a white cartoon; ligands are depicted as blue sticks.
